# Supplementary material for: Measurement-induced entanglement and teleportation on a noisy quantum processor
Source: Nature. 2023 Oct 18;622(7983):481–6. doi: 10.1038/s41586-023-06505-7 (PMC10584681; doi:10.1038/s41586-023-06505-7)
Supplement: Supplementary file 1 — Supplementary Information [file 41586_2023_6505_MOESM1_ESM.pdf]

nature portfolio

https://doi.org/10.1038/s41586-023-06505-7

Supplementary information

Measurement-induced entanglement and teleportation on a noisy quantum processor

In the format provided by the authors and unedited

Supplement - Measurement-induced entanglement and teleportation on a noisy quantum processor

Google Quantum AI and Collaborators
(Dated: July 5, 2023)

Contents

I. Quantum processor details 2
A. Coherence times and readout error 2
B. Single and two qubit errors 2

II. 2-Qubit fSim gates 2
A. fSim implementation 2
B. Characterizing leakage errors 3
C. fSim gate calibration 4

III. Measuring entropy via randomized measurements 6
A. Randomized measurements 6
B. Entropy error mitigation and additional data and numerics 6
C. Theoretical justification of error mitigation prescription 7
D. Limitations of error mitigation: highly-entangled states 10

IV. Space-time dual of fSim gates 12

V. Mapping of $2+1$-dimensional shallow circuits to $1+1$-dimensional monitored circuits 14

VI. Decoding protocol 15
A. Review 15
B. Protocol 15
C. Classical simulation algorithm 16
D. Simulated decoding data 18

VII. Approximate decoding 20
A. MPS method 20
B. Results 20
C. Effect of classical simulation errors 20

References 22

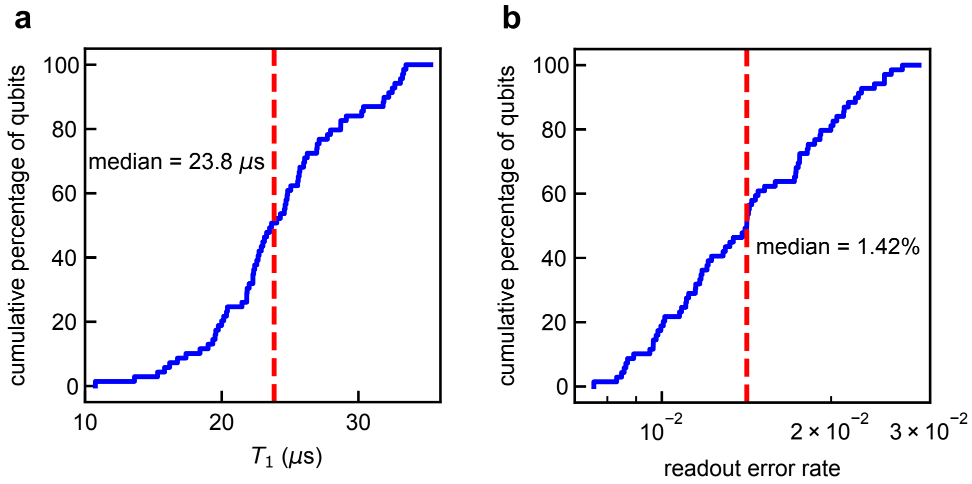

FIG. S1. **a**, Cumulative distribution of T_1 across the 70 qubit superconducting quantum processor with a dashed line indicating a median value of 23.8 μs. **b**, Cumulative distribution of the readout error across the 70 qubit superconducting quantum processor with a dashed line indicating the median value of 1.42%.

I. Quantum processor details

A. Coherence times and readout error

The experiment is performed on a quantum processor with 70 Transmon qubits with tunable frequencies and interqubit couplings with a similar design to Ref. [1]. In Fig. S1**a** we show the characteristic relaxation (T_1) times of the entire 70 qubit chip with a median of 23.8 μs, as measured through simultaneous population decay ($|1\rangle \rightarrow |0\rangle$) experiments.

We also benchmark the readout errors of the entire 70 qubit chip, which are especially important to minimize to study measurement-induced physics. All qubits are repeatedly and simultaneously prepared in a random bit string state and then readout. For each qubit, an error occurs if the resulting bit does not match its initial state. We achieve a median single qubit readout error rate of 1.42% (Fig. S1**b**).

B. Single and two qubit errors

We benchmark the error rates of the single qubit and two qubit iSWAP-like gates used in the experiment using cross entropy benchmarking (XEB)[1]. In Fig. S2 we show the cumulative distributions of single-qubit and two qubit cycle Pauli error rates, with medians of 0.10% and 0.60%, respectively. The error of a cycle corresponds to the error of two randomly chosen single-qubit gate on each qubit followed by the iSWAP-like gate.

II. 2-Qubit fSim gates

In the following sections we describe the implementation of arbitrary 2-qubit fSim gates with superconducting qubits. We then characterize the leakage errors that arise from these interactions. Finally, we discuss the specifics related to the calibration of the different fSim gates used in this experiment and their resulting fidelities.

A. fSim implementation

All of two qubit gates used in the 1D experiment highlighted in Fig. 2 in the main text are implemented as general fermionic simulation unitaries of the form

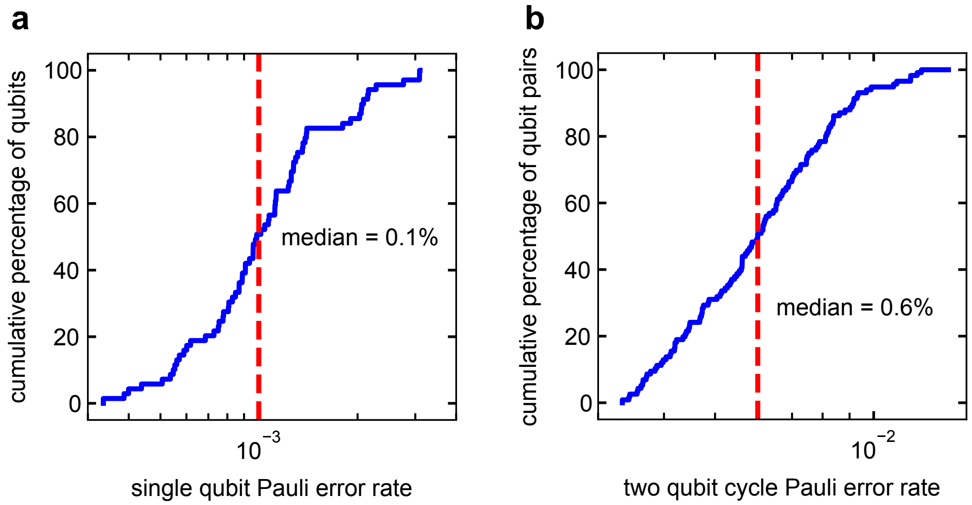

FIG. S2. **a**, **b**, Cumulative distributions of the single-qubit Pauli error (**a**) and two-qubit cycle iSWAP-like Pauli error (**b**) across the 70 qubit superconducting quantum processor as measured by XEB. We find median values of 0.10% and 0.60% for the single-qubit and iSWAP-like total cycle errors, respectively.

$$\mathrm{fSim}(\theta,\phi,\Delta_+,\Delta_-,\Delta_{-,\mathrm{off}}) = \begin{pmatrix} 1 & 0 & 0 & 0 \\ 0 & e^{i(\Delta_+ + \Delta_-)}\cos\theta & -ie^{i(\Delta_+ - \Delta_{-,\mathrm{off}})}\sin\theta & 0 \\ 0 & -ie^{i(\Delta_+ + \Delta_{-,\mathrm{off}})}\sin\theta & e^{i(\Delta_+ - \Delta_-)}\cos\theta & 0 \\ 0 & 0 & 0 & e^{i(2\Delta_+ - \phi)} \end{pmatrix}, \tag{S1}$$

where θ is the SWAP angle, ϕ is the conditional phase, and the Δ's are single qubit phases. The general pulse shape used to implement arbitrary fSim gates of this form is displayed in Fig. S3**a**. The pulse shape is defined by the maximum coupling strength g_{max}, the hold time at g_{max}, t_p, and the rise time t_{rise}. During the interaction the fundamental frequencies f_0 and f_1 of the two qubits are brought into resonance. During the time the coupler is on $(2t_{\mathrm{rise}} + t_p)$, resonant interactions between $|01\rangle$ and $|10\rangle$ of the two qubits lead to a finite value of θ, while dispersive interactions between $|11\rangle$ and $|02\rangle$ (and $|20\rangle$) lead to a finite ϕ. In terms of the coupler pulse parameters, both θ and ϕ scale linearly with t_p while $\theta \propto g_{\mathrm{max}}$ and $\phi \propto g_{\mathrm{max}}^2$. This difference in scaling enables independent control of θ and ϕ. The single qubit phases occur due to the frequency detunings of qubits during the DC pulse and are typically calibrated and set to near zero. We show typical maps of experimentally measured θ and ϕ via unitary tomography as a function of t_p and g_{max}, with $t_{\mathrm{rise}} = 5$ ns (Fig. S3**c, d**) and 8 ns (Fig. S3**e, f**). This implementation of the fSim gate is built off of the technique introduced in Ref.[2], where further details can be found.

B. Characterizing leakage errors

For an arbitary fSim gate, the above mentioned interactions with states outside the computational subspace naturally lead to leakage errors. Due to decoherence effects it is desirable to implement fast coupler pulses, while longer ones are favorable for suppressing leakage error. Furthermore, leakage become more important sources of errors as g_{max} increases (Fig. S4). Although the leakage oscillates as a function of t_p and one could carefully select the coupler gate's pulse parameters to sit at one of the leakage minima, this does not allow for precise control of the resulting SWAP angle θ and conditional phase ϕ; if the desired (θ,ϕ) happen to lie on top of a leakage maxima, the resulting fidelity of the gate will suffer. Therefore if precise control of (θ,ϕ) is needed, an alternative strategy is required to minimize leakage, particularly at large g_{max}.

In Ref. [2] leakage errors were minimized by implementing a trapezoidal coupler pulse with t_{rise} set to 5 ns. Indeed this was sufficient for realizing relatively high fidelity (99%) fSim gates for a select few (θ,ϕ), however further characterizations are needed for arbitrary (θ,ϕ). To investigate the effect of the rise time on leakage rates, we performed a systematic study of leakage as a function of g_{max}, t_p, and t_{rise}. To measure leakage, qubit pairs are initialized in the $|11\rangle$ state, allowed to interact via the fSim unitary defined by the pulse sequence in Fig. S3**a**, and then read out. Here, we measure leakage as the probability of finding the qubits in one of the leakage states ($|02\rangle$ or $|20\rangle$) after interacting via the fSim pulse, similar to Ref. [3]. In Fig. S4**a** we show maps of the leakage at fixed values of t_{rise} for $0 < g_{\mathrm{max}} < 80$ MHz and $0 < t_p < 20$ ns. As can be seen, leakage is generally suppressed as t_{rise} increases, which sets the ramp rate of the coupler pulse for a given g_{max} and the degree of "adiabaticity" of the gate. This can also be seen by plotting

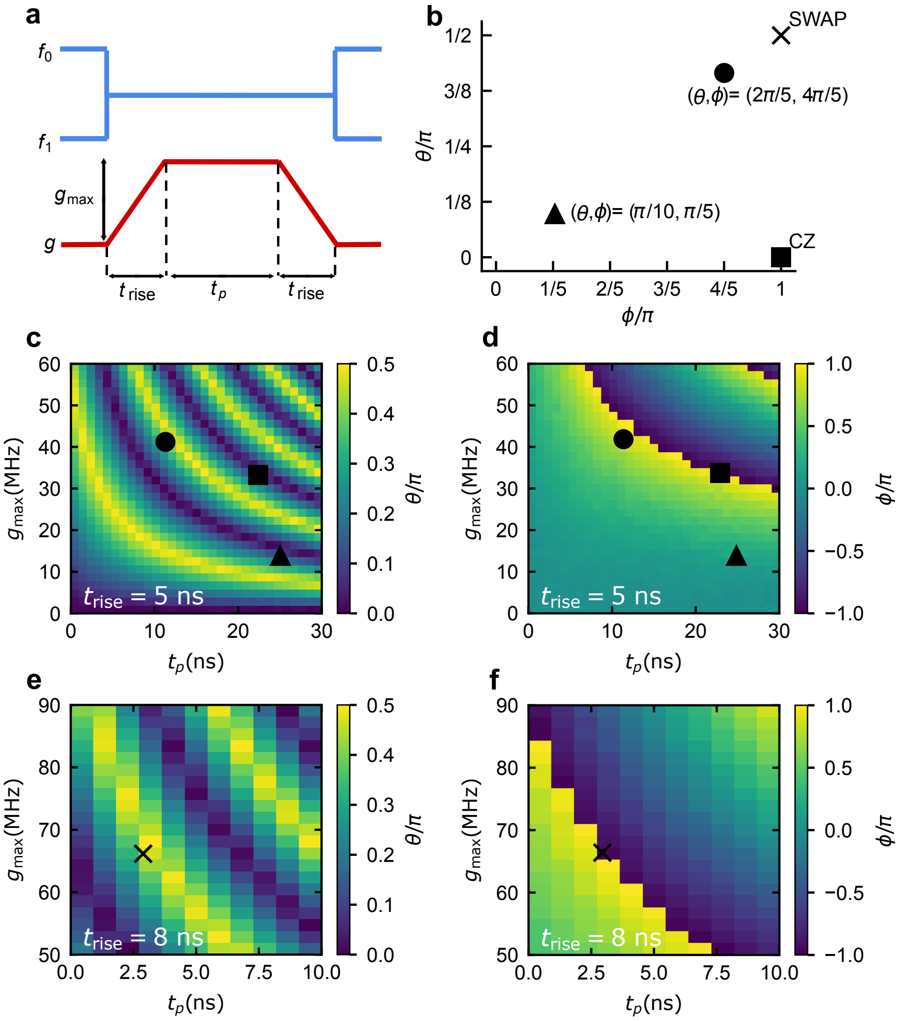

FIG. S3. **a**, Schematic illustration of the DC pulse used to implement arbitrary fSim gates. **b,** Schematic plot showing the four sets of SWAP and conditional phase angles to realize the different fSim gates used in our experiment: $(\theta,\ \phi) = (2\pi/5,\ 4\pi/5)$ (circle), $(\pi/10,\ \pi/5)$ (triangle), $(0,\ \pi)$ (CZ, square), and $(\pi/2,\ \pi)$ (SWAP, cross). **c, d,** Experimentally measured values of θ and ϕ as a function of the hold time t_p and maximum coupling strength $g_{\rm max}$ for $t_{\rm rise} = 5$ ns. The approximate pulse parameters for the fSim$(2\pi/5,\ 4\pi/5)$, fSim$(\pi/10,\ \pi/5)$, and CZ gates are overlaid on top. **e, f,** Same as **c, d,** but for $t_{\rm rise} = 8$ ns. The approximate pulse parameters for the SWAP gate is overlaid on top.

the leakage, averaged over $0 < t_p < 20$ ns, as a function of $g_{\rm max}$ for various $t_{\rm rise}$ (Fig. S4**b**). The fact that the leakage depends predominately on the ramp rate $g_{\rm max}/t_{\rm rise}$, rather than the set rise time, is illustrated in Fig. S4**c**, where the time averaged leakage for each $t_{\rm rise}$ collapses onto the same curve as a function of the ramp rate. While this eliminates the knowledge that leakage indeed oscillates as a function of t_p, it highlights a crossover from an approximately ramp rate-independent leakage behavior to one in which the leakage increases approximately proportionally with the ramp rate.

C. fSim gate calibration

In our experiment we implemented four different fSim gates: fSim$(\theta = 2\pi/5,\ \phi = 4\pi/5)$, fSim$(\theta = \pi/10,\ \phi = \pi/5)$, CZ = fSim$(\theta = 0,\ \phi = \pi)$, and SWAP = fSim$(\theta = \pi/2,\ \phi = \pi,\ \Delta_+ = \pi/2)$, where all of the Δ angles are set to 0 unless explicitly stated otherwise. In previous works the SWAP was implemented by concatenating several single qubit and two qubit gates in series [4–6]. While this works in theory, the SWAP as a composite gate results in poor error rates and a resulting long gate duration, which is undesirable for decoherence effects. A better, alternative approach is to implement SWAP with a single coupler pulse using our fSim framework. Following the analysis of the leakage, we found that $t_{\rm rise} = 8$ ns was sufficient for minimizing its leakage at large $g_{\rm max}$, and accordingly set $t_{\rm rise} = 8$ ns for the calibration of the SWAP gate, while $t_{\rm rise} = 5$ ns was set for the others. The approximate coordinates of the four fSim

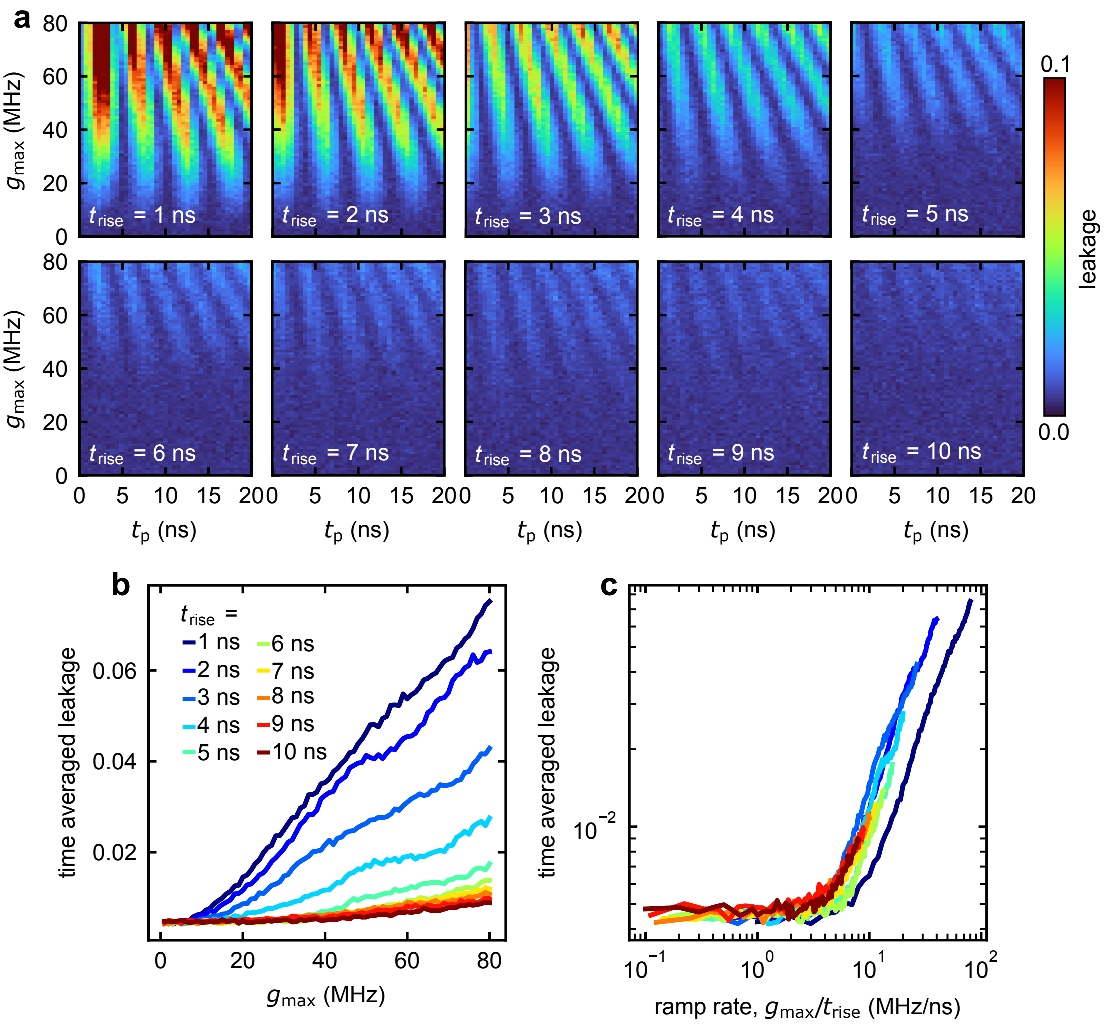

FIG. S4. **a**, Leakage measured as a function of the coupling strength $g_{\max}$ and hold time t_p for fixed values of the rise time $t_{\rm rise}$. **b,** The time averaged leakage (averaged over the range $0 < t_p < 20$ ns) as a function of $g_{\max}$ for different values of $t_{\rm rise}$. **c,** The time averaged leakage plotted as a function of the ramp rate $g_{\max}/t_{\rm rise}$ for different $t_{\rm rise}$.

gates are overlaid on top of the $(\theta,\,\phi)$ maps in Fig. S3. The target angles are calibrated by running higher resolution, local unitary tomography maps $\theta(t_p, g_{\max})$ and $\phi(t_p, g_{\max})$ near an initial guess based on the maps shown in Fig. S3. The optimal pulse parameters for target angles $(\theta_0,\,\phi_0)$ are then found by minimizing the cost function

$$f(\theta_0, \phi_0, t_p, g_{\max}) = \log[(\theta_0 - \theta(t_p, g_{\max}))^2 + (\phi_0 - \phi(t_p, g_{\max}))^2]. \tag{S2}$$

The remaining single-qubit angles are set to 0, except for SWAP where $\Delta_+ = \pi/2$. The resulting total cycle Pauli error rates of the qubit pairs that utilized each fSim gate for the experiment highlighted in Fig. 2 of the main text are displayed in Fig. S5. We achieve median cycle Pauli error rates of 0.89%, 0.98%, 1.33%, and 1.23% for fSim($2\pi/5$, $4\pi/5$), fSim($\pi/10$, $\pi/5$), CZ, and SWAP gates, respectively as measured by XEB.

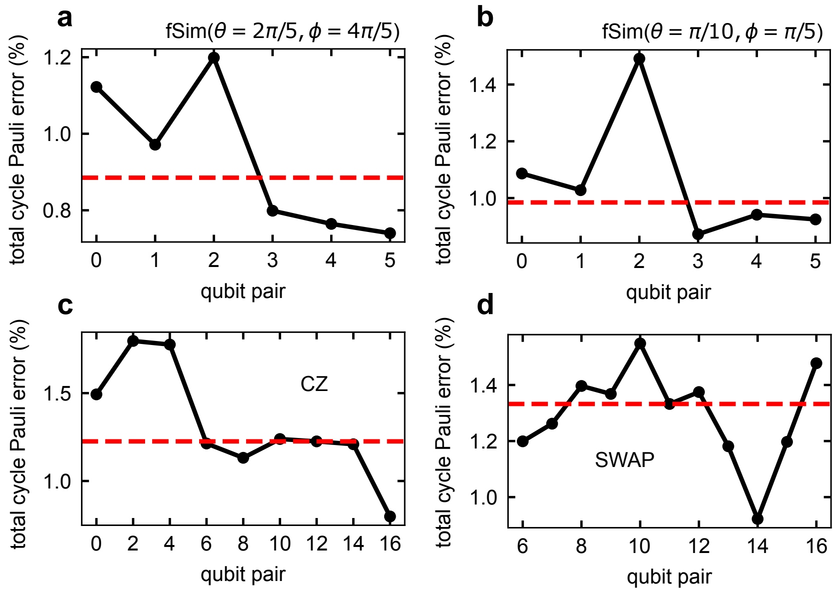

FIG. S5. **a-d**, Cycle Pauli error of the fSim($2\pi/5$, $4\pi/5$), fSim($\pi/10$, $\pi/5$), CZ, and SWAP gates, respectively, as measured by cross entropy benchmarking (XEB) for the qubit pairs that utilized each gate. Red dashed line corresponds to the median.

III. Measuring entropy via randomized measurements

A. Randomized measurements

In our experiments we utilize the randomized measurement protocol [7–10] to measure the second Renyi entropy. For N qubits, the protocol relies on applying random single qubit unitaries $U = u_1 \otimes u_2 \otimes ... \otimes u_N$, each independently drawn from the circular unitary ensemble (CUE). For a system A of $N_A \leq N$ qubits, the purity is given by

$$\mathrm{Tr}(\rho_A^2) = 2^{N_A} \sum_{s,s'} (-2)^{-H(s,s')} \langle P(s)P(s') \rangle_U, \tag{S3}$$

where ρ_A is the density matrix of A, $H(s, s')$ is the Hamming distance between binary bitstrings s and s' in the computational basis and $P(s)$ is the probability of measuring s. The average over probabilities of bitstrings $\langle P(s)P(s') \rangle_U$ is averaged over several instances of U and in practice is estimated with jackknife resampling to remove bias. The second Renyi entropy is then given by $S_A^{(2)} = -\log_2(\mathrm{Tr}(\rho_A^2))$. A particular advantage of the randomized measurement method is that the same set of measurements can be used to simultaneously calculate the entropies for multiple subsystems. This is especially beneficial for calculating the second Renyi mutual information $I_{AB}^{(2)} = S_A^{(2)} + S_B^{(2)} - S_{AB}^{(2)}$. Previous successful implementations of the technique can be found in Refs. [9, 10].

B. Entropy error mitigation and additional data and numerics

In today's quantum processors noise is pervasive and unavoidable. Since noise adds a background entropy, it can lead to important deviations from theoretically predicted values. Consequently, it is essential to account for and mitigate the effects of noise when measuring entropy in an experiment. Our method for error mitigating noise relies on the fact that we perform randomized measurements of entropy on the entirety of a 1D chain that is expected to be in a pure state; any entropy of the global state of the chain must come solely from noise contributions. We further assume that background entropy due to noise is extensive and with a spatially-uniform density, such that for any subsystem A of the pure state AB, the background entropy δS is given by

$$\delta S(A) = \frac{\mathrm{vol}(A)}{\mathrm{vol}(AB)} \delta S(AB). \tag{S4}$$

In Fig. S6**a** we show the raw, non-error-mitigated entropy curves for the two examples of a 18 qubit space-time dual circuit. For each curve, bitstrings are collected over 20 random instances (different random choices of single qubit gates)

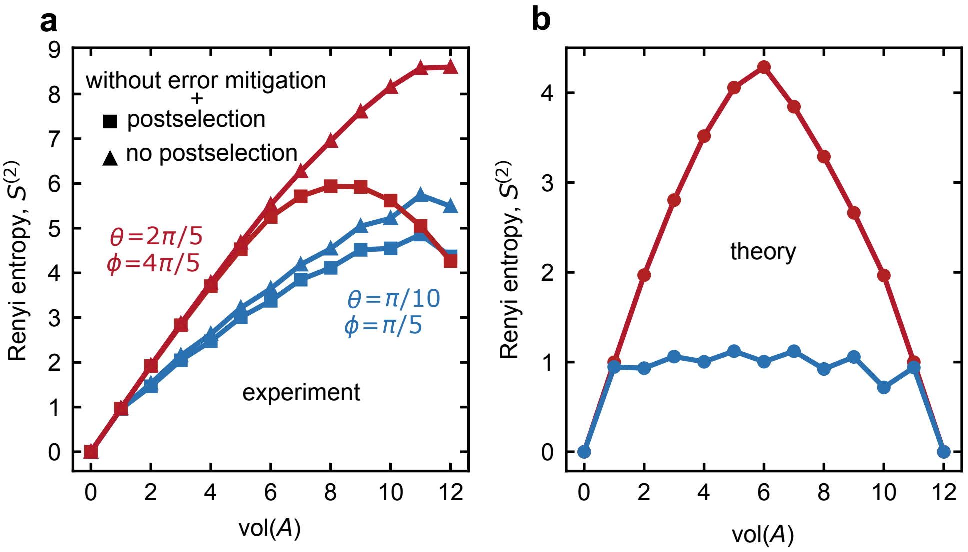

FIG. S6. **a**, Non error-mitigated Renyi entropy as a function of the volume of a subsystem A when postselecting (squares) and not postselecting (triangles) on the measurement record of qubits Q_1, Q_2, Q_3, Q_4, Q_5, and Q_6 for the experiment implemented in Fig. 2 of the main text. **b**, Results of an exact numerical simulation of the second Renyi entropy as a function of the volume of a subsystem A for the experiment implemented in Fig. 2 of the main text. The curves agree qualitatively well with the experimental results.

of the circuit with 5 instances of CUE unitaries U each and 10 million shots per instance of U. We then postselect on the most probable measurement outcome of the 6 measured qubits and use the outcome of the randomized measurements to extract the second Renyi entropy of subsystems of the 12 remaining qubits. Each data point corresponds to an average over all contiguous subsystems of the specified volume. There is a significant residual entropy for the state of the whole system due to noise, which grows both with the size of the chain and the depth of the circuit. We then apply our error mitigation technique by subtracting a straight line that intersects the origin and the whole-system ($\mathrm{vol}(A) = 12$) entropy. In Fig. S6**b** we show exact numerical simulations of the second Renyi entropy, which show good qualitative agreement with the experiment.

We apply this same strategy for the randomized measurements of our experiment on a 2D grid of 19 qubits. In Fig. S7**a** we show the experimental results without error mitigation as a function of the circuit depth T. For each curve, bitstrings are collected over 20 random instances (different random choices of single qubit gates) of the circuit with 5 instances of CUE unitaries U each and 20 million shots per instance of U. We then postselect on the most probable measurement outcome of the 12 measured qubits and use the outcome of the randomized measurements to extract the second Renyi entropy of subsystems of the 7 remaining qubits. Each data point corresponds to an average over all contiguous subsystems of the specified volume. As expected, the whole-system entropy ($\mathrm{vol}(A) = 7$) grows approximately linearly with circuit depth (Fig. S7**b**). Noticeably, the whole-system entropy intersects the y-axis at a finite value of δS. We attribute this residual background entropy to readout error, which remains an approximately constant source of error as a function of circuit depth. We note that this residual entropy due to readout error comes both from error in the readout of the 12 postselected qubits and of the remaining chain of 7 qubits whose entropy we obtain via randomized measurements. The results for an exact numerical simulation (Fig. S7**c**) agrees qualitatively with the experiment.

We also present additional data complementary to Fig. 3 of the main text. In Fig. S8 we plot the second Renyi mutual information $\mathcal{I}_{AB}^{(2)}$ as a function of the total volume of the combined subsystems A and B of the 7 edge qubits. When $T \geq 4$, $\mathcal{I}_{AB}^{(2)}$ is large if A and B together make up over half the system (≥ 4 qubits) and small otherwise (≤ 3 qubits), consistent with the expected behavior of highly entangled states. Lastly, we again highlight the importance of postselection. In Fig. S9 we show the equivalent results to Fig. 3 of the main text, but without postselection. We find no signature of a transition between entanglement phases in the 1D chain of 7 qubits and correlations between qubits, as measured by the second Renyi mutual information, are entirely absent.

C. Theoretical justification of error mitigation prescription

Here we show that the prescription in Eq. (S4) is theoretically justified for states prepared by short time evolution. Specifically, we show that, to leading order in the noise strength ϵ, the entropy $S_A^{(2)}$ incurs an extensive error $\sim \epsilon N_A$

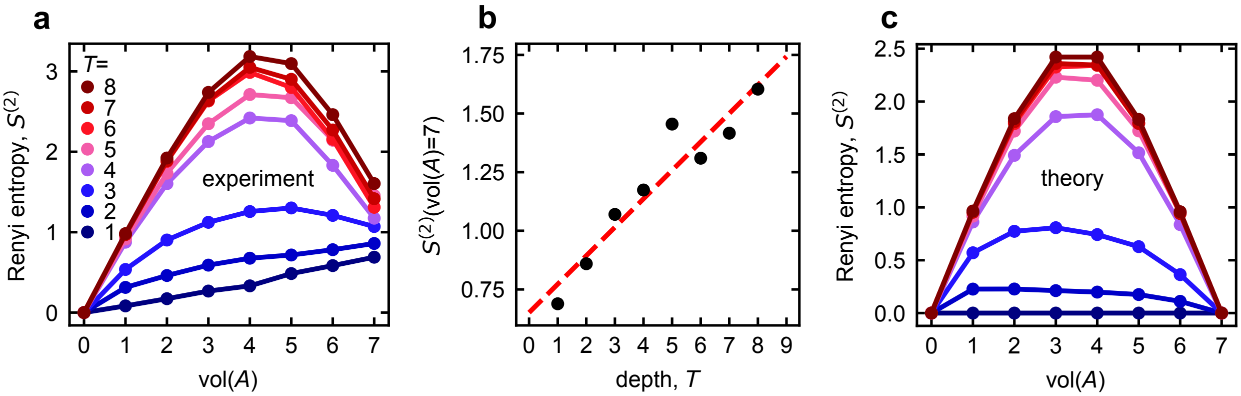

FIG. S7. **a**, Experimentally measured second Renyi entropy as a function of a sub-system A of the chain of 7 qubits at various depths without error mitigation. **b**, Second Renyi entropy of the entire 7 qubit chain after measurement of the lower 12 qubits as a function of circuit depth. The entropy grows approximately linearly due to decoherence in the system. Dashed line is a linear fit and intercepts the y-axis at a non-zero value, which we attribute to readout error. **c**, Exact numerical simulation of the second Renyi entropy as a function of the volume of a sub-system A of the 7 edge qubits at the same depths as in **a**. The results agree qualitatively well with the error-mitigated data presented in the main text.

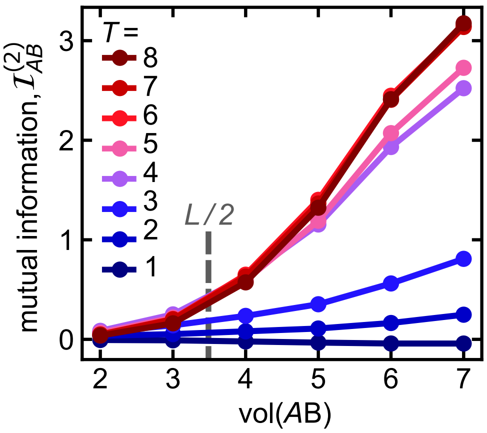

FIG. S8. Second Renyi mutual information $\mathcal{I}^{(2)}_{AB}$ as a function of the combined volume of A and B for each T. Each point is averaged over all possible configurations of contiguous subsystems A and B for a fixed total volume (i.e. averaged over all values of x between A and B).

(where N_A is the number of qubits in A), while the mitigated entropy incurs only a constant error, $\sim$ const. $\times\,\epsilon$.

Let us for simplicity consider a quantum state $|\psi\rangle$ produced by the ideal, noiseless circuit, and affected by a local noise channel $\mathcal{E}$ only at the end of the dynamics (this approximation is reasonable as long as we focus on states prepared by short unitary evolution, where a large fraction of the noise comes from measurement error and even mid-circuit gate error may be commuted to the final time at the expense of finite-range correlations). The randomized measurement protocol probes the entropy of the noisy state $\rho_A^{\text{noisy}} = \text{Tr}_{\bar{A}}[\mathcal{E}^{\otimes N}(|\psi\rangle\langle\psi|)]$, while we aim to recover the entropy of the ideal state $\rho_A = \text{Tr}_{\bar{A}}[|\psi\rangle\langle\psi|]$. Note that $\rho_A^{\text{noisy}} = \mathcal{E}^{\otimes N_A}(\rho_A)$, where N_A is the number of qubits in A, due to $\mathcal{E}$ being trace-preserving.

We take the local noise channel $\mathcal{E}$ to be single-qubit depolarizing noise, which is often a good approximation in the context of random circuit dynamics [1]:

$$\mathcal{E}_i(\rho_A) = e^{\epsilon\mathcal{L}_i}(\rho_A), \quad \mathcal{L}_i(\rho_A) = -3\rho_A + \sum_{\alpha=x,y,z} \sigma_i^\alpha \rho_A \sigma_i^\alpha = -4\rho_A + 2I_i \otimes \rho_{A\backslash\{i\}}. \tag{S5}$$

Here ϵ parametrizes the noise strength and $\mathcal{L}_i$ is the Liouvillian generator of the noise channel, in the Gorini-Kossakowski-Sudarshan-Lindblad form with jump operators $\{\sigma_i^\alpha : \alpha = x, y, z\}$. We have additionally exploited the decomposition of the erasure channel $\frac{1}{2}I_i \otimes \rho_{A\backslash\{i\}} = \frac{1}{4}\left(\rho_A + \sum_{\alpha=x,y,z}\sigma_i^\alpha\rho_A\sigma_i^\alpha\right)$ in the second equality.

The entropy mitigation prescription Eq. (S4) is to approximate the ideal entropy $S_A^{(2)} = -\log_2 \text{Tr}\left(\rho_A^2\right)$ by the quantity

$$\tilde{S}_A^{(2)} = -\log_2 \text{Tr}\left[(\rho_A^{\text{noisy}})^2\right] + \frac{N_A}{N}\log_2 \text{Tr}\left[(\rho^{\text{noisy}})^2\right]. \tag{S6}$$

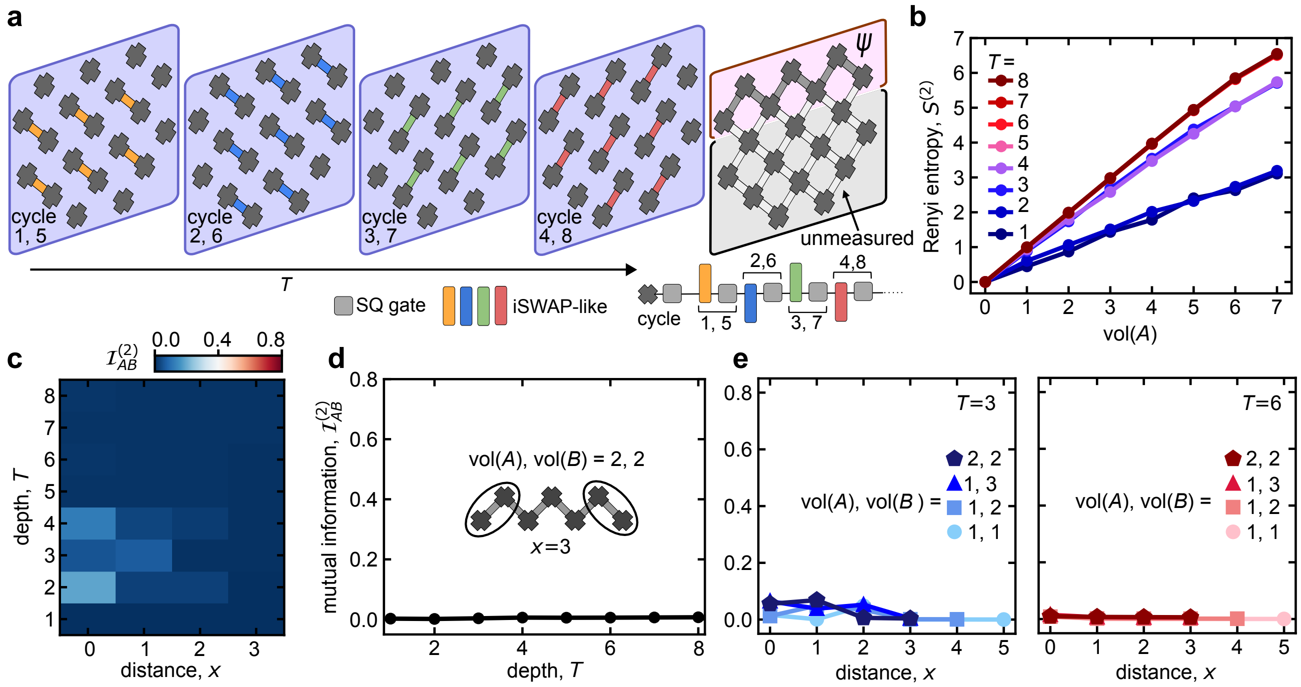

FIG. S9. **a**, Schematic of the 2D grid of qubits. At each cycle of the circuit, random single qubit and two-qubit iSWAP-like gates are applied to each qubit in the cycle sequence shown. At the end of the circuit, the measurement outcomes of the 12 lower M qubits are *not* postselected on. **b**, Second Renyi entropy of contiguous subsystems A of the $L = 7$ edge qubits at various depths. The measurement is not noise-mitigated here. **c**, Second Renyi mutual information $\mathcal{I}_{AB}^{(2)}$ between 2-qubit subsystems A and B vs depth T and distance x (number of qubits between A and B). **d**, $\mathcal{I}_{AB}^{(2)}$ as a function of T for 2-qubit subsystems A and B at maximum separation. **e**, **f**, $\mathcal{I}_{AB}^{(2)}$ vs. x for $T = 3$ and $T = 6$, respectively, for different volumes of A and B.

To evaluate the effect of weak noise on the mitigated entropy, we differentiate with respect to ϵ at $\epsilon = 0$:

$$\begin{aligned}\frac{d}{d\epsilon}\tilde{S}_A^{(2)}\Big|_{\epsilon=0} &= -\frac{2}{\ln(2)}\sum_{i=1}^{N_A}\frac{\mathrm{Tr}[\rho_A\mathcal{L}_i(\rho_A)]}{\mathrm{Tr}\,\rho_A^2} + \frac{2N_A}{\ln(2)N}\sum_{i=1}^{N}\frac{\mathrm{Tr}[\rho\mathcal{L}_i(\rho)]}{\mathrm{Tr}\,\rho^2} \\ &= \frac{4N_A}{\ln(2)}\left\{\frac{1}{N_A}\sum_{i=1}^{N_A}\left[2-\frac{\mathrm{Tr}(\rho_{A\setminus\{i\}}^2)}{\mathrm{Tr}\,\rho_A^2}\right] - \frac{1}{N}\sum_{i=1}^{N}\left[2-\mathrm{Tr}(\rho_{\{i\}}^2)\right]\right\} \\ &= -\frac{4N_A}{\ln(2)}\left\{\frac{1}{N_A}\sum_{i=1}^{N_A}\mathrm{Tr}\left(\rho_{\{i\}}^2\right)2^{\mathcal{I}_{\bar{A},\{i\}}^{(2)}} - \frac{1}{N}\sum_{i=1}^{N}\mathrm{Tr}(\rho_{\{i\}}^2)\right\}\end{aligned} \tag{S7}$$

In the second line we have used the form of $\mathcal{L}_i$ from Eq. (S5) and the fact that $\rho = |\psi\rangle\langle\psi|$ is pure (note the expression is evaluated at $\epsilon = 0$) to simplify $\mathrm{Tr}\,\rho^2 = 1$ and $\mathrm{Tr}\,\rho_{\overline{\{i\}}}^2 = \mathrm{Tr}\,\rho_{\{i\}}^2$. In the third line we have used the definition of Renyi-2 mutual information, and again the fact that ρ as a whole is pure, to simplify

$$\frac{\mathrm{Tr}\left(\rho_{A\setminus\{i\}}^2\right)}{\mathrm{Tr}\,\rho_A^2} = \mathrm{Tr}\left(\rho_{\{i\}}^2\right)\left(\frac{\mathrm{Tr}\left(\rho_{\bar{A}\cup\{i\}}^2\right)}{\mathrm{Tr}\,\rho_{\{i\}}^2\,\mathrm{Tr}\,\rho_{\bar{A}}^2}\right) = \mathrm{Tr}\left(\rho_{\{i\}}^2\right)2^{S_{\{i\}}^{(2)}+S_{\bar{A}}^{(2)}-S_{\bar{A}\cup\{i\}}^{(2)}} = \mathrm{Tr}\left(\rho_{\{i\}}^2\right)2^{\mathcal{I}_{\bar{A},\{i\}}^{(2)}}. \tag{S8}$$

Finally, neglecting the position dependence of single-site purities and setting $\mathrm{Tr}\,\rho_{\{i\}}^2 \equiv f \in [1/2, 1]$ for all i, we arrive at

$$\frac{d}{d\epsilon}\tilde{S}_A^{(2)}\Big|_{\epsilon=0} = \frac{4f}{\ln(2)}\sum_{i\in A}\left(2^{\mathcal{I}_{\bar{A},\{i\}}^{(2)}}-1\right) \approx 4f\sum_{i\in A}\mathcal{I}_{\bar{A},\{i\}}^{(2)}. \tag{S9}$$

The approximation holds to leading order in small mutual information. Clearly for the case of unitary dynamics the mutual information is short-ranged, being bounded by the light cone. Even in the presence of measurements, a general stability condition requires the sum to remain finite in the limit of large A in 1D systems [11]. Thus the error-mitigated entropy carries a *finite* error

$$\tilde{S}_A^{(2)}\Big|_{\epsilon} - S_A^{(2)}\Big|_{\epsilon=0} = C\epsilon + O(\epsilon^2) \tag{S10}$$

for small noise strength ϵ, where C is a constant. At the same time it is apparent from Eq. (S7) that the un-mitigated entropy carries an extensive error

$$S_A^{(2)}\Big|_\epsilon - S_A^{(2)}\Big|_{\epsilon=0} = C' N_A \epsilon + C'' \epsilon + O(\epsilon^2) \tag{S11}$$

where C', C'' are constants. Thus, while the error mitigation prescription does not completely reproduce the ideal, noiseless behavior, in the case of states prepared by short time evolution (whether unitary or monitored) it does remove the dominant entropy density contribution coming from noise, and in particular allows the correct identification of area-law states.

D. Limitations of error mitigation: highly-entangled states

Here we complement the analysis above by illustrating an example of how the error mitigation may fail in highly-entangled states by producing significant distortions of the underlying noiseless behavior. As an extreme example, one may consider a Haar-random state $|\psi\rangle$ on a bipartite system AB and compute the purity $\mathrm{Tr}\left[(\rho_A^{\mathrm{noisy}})^2\right]$ after the state has been subjected to local depolarizing noise. Formally we may write this quantity as a matrix element of the noise super-operator:

$$2^{-S_A^{(2)}} = \mathrm{Tr}\left[(\rho_A^{\mathrm{noisy}})^2\right] = (\chi_A e_B | \mathcal{E}^{\otimes 2N} | \rho^{\otimes 2}) \tag{S12}$$

where $\rho = |\psi\rangle\langle\psi|$ is the pure Haar-random state, $\mathcal{E}$ is a single-qubit depolarizing channel as before, and e, χ are permutation operators acting on the two replicas of the Hilbert space: $e\,|i\rangle_1\,|j\rangle_2 = |i\rangle_1\,|j\rangle_2$ (identity permutation) and $\chi\,|i\rangle_1\,|j\rangle_2 = |j\rangle_1\,|i\rangle_2$ (transposition). Furthermore we use the notation $|O)$ to represent an operator O as a vector in a doubled Hilbert space, with inner product $(O|P) = \mathrm{Tr}(O^\dagger P)$.

The purity can be exactly averaged over Haar-random states by using the fact that

$$\mathbb{E}_{\psi\sim\mathrm{Haar}}[|\psi\rangle\langle\psi|^{\otimes 2}] = \frac{e_A e_B + \chi_A \chi_B}{2^N(2^N+1)}. \tag{S13}$$

Next, we note that the noise super-operator $\mathcal{E}^{\otimes 2}$ leaves invariant both the "super-ket" $|e)$ (unitality of the depolarizing channel, $\mathcal{E}(I) = I$) and the "super-bra" $(e|$ (trace preservation, $\mathcal{E}^*(I) = I$). Using the inner product rules $(e|e) = (\chi|\chi) = d^2$ and $(e|\chi) = (\chi|e) = d$ where d is the relevant Hilbert space dimension, this gives

$$\begin{aligned}
\mathbb{E}_{\psi\sim\mathrm{Haar}}\left[2^{-S_A^{(2)}}\right] &= \frac{(\chi_A e_B|\mathcal{E}^{\otimes 2N}|e_A e_B) + (\chi_A e_B|\mathcal{E}^{\otimes 2N}|\chi_A \chi_B)}{2^N(2^N+1)} \\
&= \frac{2^{2N_B}(\chi_A|e_A) + 2^{N_B}(\chi_A|\mathcal{E}^{\otimes 2N_A}|\chi_A)}{2^N(2^N+1)} \\
&= \frac{2^{N_B} + 2^{-N_A}(\chi_i|\mathcal{E}_i^{\otimes 2}|\chi_i)^{N_A}}{2^N+1}.
\end{aligned} \tag{S14}$$

In the last line we have used the fact that $\chi_A = \bigotimes_{i\in A}\chi_i$ to factor the matrix element $(\chi_A|\mathcal{E}^{\otimes 2N_A}|\chi_A)$ into on-site matrix elements. It is now helpful to decompose $\chi_i = (I^{\otimes 2} + X^{\otimes 2} + Y^{\otimes 2} + Z^{\otimes 2})/2$ and note that $\mathcal{E}(I) = I$ while $\mathcal{E}(X) = e^{-\epsilon}X$, and the same for Y and Z. Thus $(\chi_i|\mathcal{E}_i^{\otimes 2}|\chi_i) = 1 + 3e^{-2\epsilon}$, and we conclude

$$\mathbb{E}_{\psi\sim\mathrm{Haar}}\left[2^{-S_A^{(2)}}\right] = \frac{2^{N_B} + [(1+3e^{-2\epsilon})/2]^{N_A}}{2^N+1}. \tag{S15}$$

Based on quantum typicality, the state-averaged result is also typical, i.e., the behavior of typical individual states from the Hilbert space should be well-described by the above formula, giving in the limit of large N

$$2^{-S_A^{(2)}} \simeq 2^{-N_A} + \left(\frac{1+3e^{-2\epsilon}}{4}\right)^{N_A} 2^{-N_B}. \tag{S16}$$

While the ascending part of the Page curve (small N_A) is nearly unchanged, the descending part is significantly affected by noise, giving in particular and entropy $S^{(2)} \simeq N \log_2[4/(1+3e^{-2\epsilon})]$ for the whole system.

Using the error mitigation prescription above, we subtract the corresponding entropy density to get

$$2^{-\tilde{S}_A^{(2)}} \simeq 2^{-N_B} + \left(\frac{2}{1+3e^{-2\epsilon}}\right)^{N_A}. \tag{S17}$$

In the mitigated entropy $\tilde{S}_A^{(2)}$, the descending part of the Page curve (small N_B) is accurately reproduced, and in particular terminates at zero entropy (for $N_B = 0$) as is expected for a pure state. However, the ascending part of the Page curve (small N_A) has an incorrect slope, $S_A^{(2)} \simeq \sigma N_A$ with "entropy density" $\sigma = \log_2[(1 + 3e^{-2\epsilon})/2] < 1$ for $\epsilon > 0$. Moreover, the maximum of $S^{(2)}$ (which is at the half-system cut $N_A = N/2$ in the absence of noise) moves to $N_A \simeq N/(1 + \sigma) \simeq N/2 + \epsilon(3/4 \ln(2))N$ (the latter valid at small ϵ). This asymmetry is qualitatively consistent with the observed behavior of our experimental error-mitigated data in Fig. 2 and 3 in the main text.

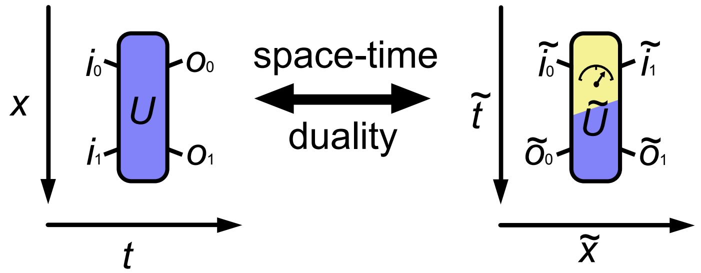

FIG. S10. Space-time dual transformation between a unitary gate U and generically non-unitary gate $\tilde{U}$. The dimensions which act as "space" and "time" are swapped through this transformation: $x \leftrightarrow \tilde{t}, t \leftrightarrow \tilde{x}$.

IV. Space-time dual of fSim gates

In Fig. 2 in the main text we use fSim gates with different parameters in order to realize different entanglement structures via space-time duality. Here we review the action of the space-time duality on fSim gates and obtain the types of measurements that appear in the effective monitored dynamics.

If i_o and i_1 label the qubit input states and o_0, o_1 the qubit output states, then the space-time duality transformation acts on a two-qubit unitary gate U as a permutation of indices, namely

$$U^{o_0,o_1}_{i_0,i_1} = \tilde{U}^{i_1,o_1}_{i_0,o_0}. \tag{S18}$$

In other words while U maps input states $i_{0,1}$ to output states $o_{0,1}$, $\tilde{U}$ maps left states i_0, o_0 to right states i_1, o_1 (Fig. S10). The linear map $\tilde{U}$ is generically non-unitary.

We focus on fSim gates with parameters (θ, ϕ) that obey $\phi = 2\theta$, which interpolate between SWAP ($\theta = \pi/2$) and identity ($\theta = 0$). The two gate sets used in the main text are $\theta = 2\pi/5$ and $\theta = \pi/10$. Using the definition in Eq. (S18), the space-time duality transformation acts on the matrix elements of the unitary gate U as follows:

$$U = \begin{pmatrix} 1 & 0 & 0 & 0 \\ 0 & \cos\theta & -i\sin\theta & 0 \\ 0 & -i\sin\theta & \cos\theta & 0 \\ 0 & 0 & 0 & e^{-2i\theta} \end{pmatrix} \mapsto \tilde{U} = \begin{pmatrix} 1 & 0 & 0 & \cos\theta \\ 0 & 0 & -i\sin\theta & 0 \\ 0 & -i\sin\theta & 0 & 0 \\ \cos\theta & 0 & 0 & e^{-2i\theta} \end{pmatrix} \tag{S19}$$

(the colors denote pairs of entries whose positions are being swapped under the transformation Eq. (S18): blue $\leftrightarrow$ red and orange $\leftrightarrow$ green).

One has $\tilde{U} = 2VH$ where V is unitary and H is Hermitian and satisfies $0 \leq H^2 \leq I$ [12]. This allows us to view H as an outcome of a generalized measurement (namely an *instrument* in a positive-operator-valued measure, e.g. $\{H^2, I - H^2\}$). From explicit calculation of $H = \sqrt{\tilde{U}^\dagger \tilde{U}}/2$ we obtain

$$H = \frac{1}{2}\sqrt{1 + 3\cos^2(\theta)}\, |\psi_\theta\rangle\langle\psi_\theta| + \frac{1}{2}|\sin(\theta)|(I - |\psi_\theta\rangle\langle\psi_\theta|) \tag{S20}$$

with $|\psi_\theta\rangle \equiv \frac{1}{\sqrt{2}}(e^{-i\theta}|00\rangle + e^{i\theta}|11\rangle)$.

As consistency checks, one can see that $\theta = \pi/2$ yields $H = I/2$, i.e. there is no measurement: $\tilde{U} = V$. This is consistent with the fact that SWAP is a self-dual gate. On the opposite end, for $\theta = 0$ ($U = I$), one gets a projector $H = |\psi_0\rangle\langle\psi_0|$ with $|\psi_0\rangle = (|00\rangle + |11\rangle)/\sqrt{2}$, a Bell pair state. Intermediate values of θ correspond to weak measurements of the operator $|\psi_\theta\rangle\langle\psi_\theta|$. The unitary V is a composition of iSWAP and a 2×2 unitary operation V' acting on the two-dimensional subspace spanned by $|00\rangle$ and $|11\rangle$. For the values of θ used in the main text, we have:

$$H = 0.1545I + 0.8090\,|\psi_{\pi/10}\rangle\langle\psi_{\pi/10}|, \qquad V' = \begin{pmatrix} 0.6487 - 0.3993i & 0.4935 + 0.4198i \\ 0.4935 + 0.4198i & 0.2901 - 0.7043i \end{pmatrix} \qquad (\theta = \pi/10), \tag{S21}$$

$$H = 0.4755I + 0.0916\,|\psi_{2\pi/5}\rangle\langle\psi_{2\pi/5}|, \qquad V' = \begin{pmatrix} 0.9585 - 0.0249i & 0.2724 + 0.0807i \\ 0.2724 + 0.0807i & -0.7901 - 0.5432i \end{pmatrix} \qquad (\theta = 2\pi/5). \tag{S22}$$

The former value gives a stronger measurement (H closer to a projector).

Finally, we verify that the two values of θ used in the main text giver rise to localized and ergodic unitary dynamics, respectively. We consider time-periodic dynamics generated by the Floquet unitary

$$U_F(\theta) = e^{-i\sum_i h_i Z_i} \bigotimes_{i \text{ even}} \text{fSim}_{i,i+1}(\theta, 2\theta) e^{-i\sum_i h_i Z_i} \bigotimes_{i \text{ odd}} \text{fSim}_{i,i+1}(\theta, 2\theta) \tag{S23}$$

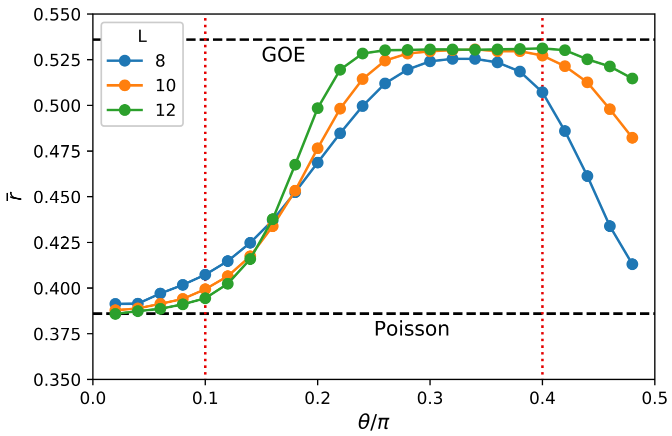

FIG. S11. Mean level spacing ratio $\bar{r}$ for the quasienergy spectrum of the Floquet operator $U_F(\theta)$, Eq. (S23), in the half-filling charge sector, as a function of fSim angle θ. Data averaged over at least 10^3 realization of disorder for each system size. Vertical dotted lines represent the values used in the main text, $\theta = 0.1\pi$ and $\theta = 0.4\pi$. Horizontal dashed lines represent the reference values for Poisson (localized) and GOE (ergodic) models.

on a 1D chain of L qubits with open boundary conditions. The h_i are random fields drawn uniformly from $[0, 2\pi)$. As the dynamics possesses a $U(1)$ symmetry (generated by $\sum_i Z_i$), the Hilbert space breaks up into decoupled charge sectors. We focus on the largest such sector, $\sum_i Z_i = 0$ (we take L even). We then diagonalize $U_F(\theta)$ restricted to this sector, obtaining eigenvalues $\{e^{-iE_n}\}$ and thus a set of *quasi-energies* $\{E_n\}$. A standard diagnostic of ergodicity is the mean level spacing ratio $\bar{r}$ defined as the average over n of

$$r_n = \frac{\min(\delta_n, \delta_{n+1})}{\max(\delta_n, \delta_{n+1})}, \quad \delta_n \equiv E_{n+1} - E_n. \tag{S24}$$

We assumed the E_n are sorted in ascending order. The quantity $\bar{r}$ takes on universal values $\bar{r} = 0.386\ldots$ in the localized phase (Poisson level statistics) and $\bar{r} = 0.536\ldots$ in the ergodic phase with time reversal symmetry (Gaussian Orthogonal Ensemble (GOE) level statistics). Fig. S11 shows numerical results for $\bar{r}$ as a function of θ for chains of $L = 8, 10, 12$ sites, showing a crossover between a localized and an ergodic regime at $\theta \simeq 0.15\pi$ at these sizes. Therefore, for the system size used in our experiment ($L = 7$, Fig.2 in the main text), the values $\theta = \pi/10$ and $\theta = 2\pi/5$ are firmly in the localized and ergodic regimes, respectively.

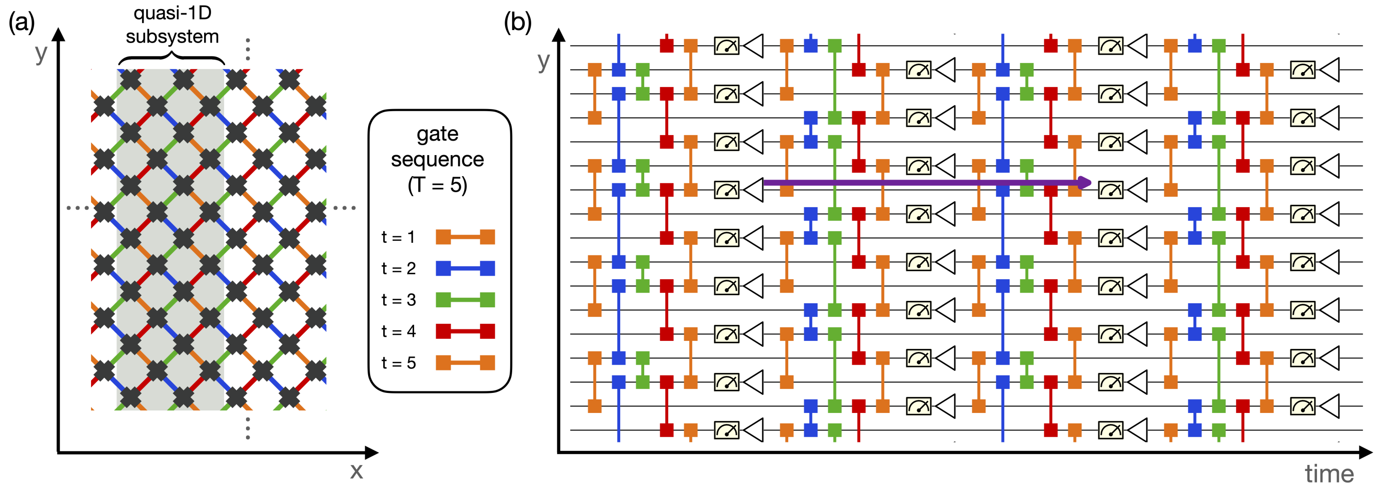

FIG. S12. Mapping of $(2+1)$-dimensional shallow circuits to $(1+1)$-dimensional monitored circuits. (a) 2D circuit architecture and gate sequence. (b) Equivalent 1D monitored circuit. Each measurement is followed by a reset to the $|0\rangle$ state (white triangles). The purple arrow represents the "life cycle" of a qubit in the dynamics, from initialization in $|0\rangle$ through the depth-5 gate sequence (orange, blue, green, red, orange), to final measurement. The circuit architecture is periodic in space and time.

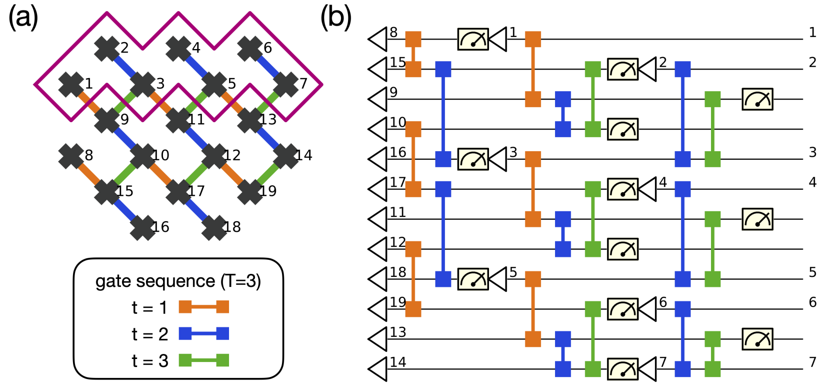

FIG. S13. Exact mapping of 2D shallow circuit used in Fig. 3 of the main text, for depth $T=3$, to 1D monitored circuit. (a) Shallow 2D circuit architecture. Qubits are numbered from 1 to 19; qubits Q_1 through Q_7 (purple contour) make up the final state. (b) Equivalent 1D monitored circuit. White triangles represent resets to $|0\rangle$ states after measurement. Numbers on each qubit wire indicate the corresponding location in the 2D circuit (panel a). The circuit acts on 12 qubits but the final state involves only 7 of them (indicated by numbers on the right), the remaining 5 are decoupled by measurements.

V. Mapping of $2+1$-dimensional shallow circuits to $1+1$-dimensional monitored circuits

Here we provide details on the mapping between $(2+1)$-dimensional shallow circuits with final measurements to $(1+1)$-dimensional dynamics with mid-circuit measurements, following Ref. [13].

We consider the circuit architecture of Fig. S12(a), which is spatially-infinite and has depth $T=5$. We take a quasi-1D system oriented along the y direction, composed of two consecutive columns in the 2D system (Fig. S12(a)). The $(2+1)$D circuit may be exactly implemented by a sequence of gates, measurements and resets on this quasi-1D system. With each step, the quasi-1D subsystem moves in the x direction, eventually "sweeping" the whole 2D lattice (this mapping is the basis of the "space-evolving block decimation" algorithm for sampling shallow circuits of Ref. [13]). The resulting $(1+1)$D circuit for our case is shown in Fig. S12(b). The spatial range of the two-qubit gates is proportional to T; for $T=5$ we find up to third-nearest-neighbor gates. This shows that the mapping is only useful for shallow circuits (deep circuits would give rise to non-local interactions).

In addition, we show in Fig. S13 a specific instance of this mapping for the 19-qubit subsystem used in Fig. 3 of the main text.

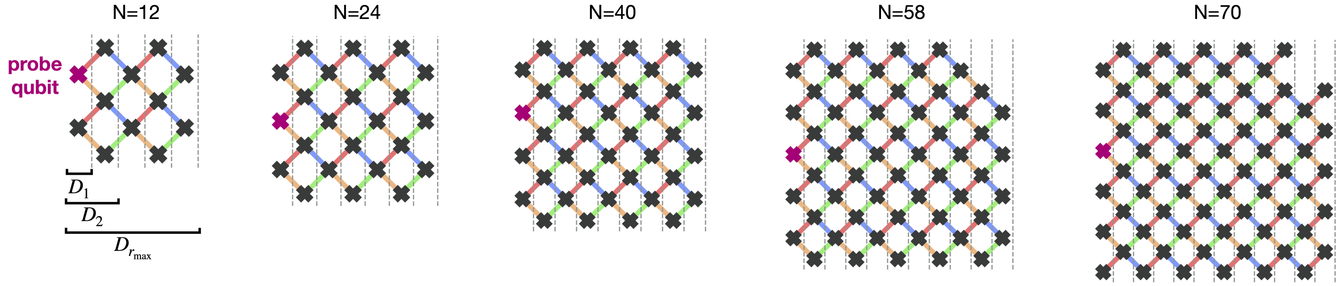

FIG. S14. The approximately square geometries of $N = 12, 24, 40, 58$, and 70 qubits used in the decoding and finite size analysis experiment of Fig. 4 in the main text. The bond colors denote the gate sequence ((orange, blue, green, red, orange). For each grid we show the probe qubit and the decoding patches $D_1, \ldots D_{r_{\max}}$ employed in the decoding algorithm (dashed lines).

VI. Decoding protocol

The data in Figure 4 (main text) is obtained by decoding the entanglement of a local probe qubit [14] on a 2D grid of $N = 12, 24, 40, 58$, and 70 qubits (Fig S14). Here we briefly review the general idea of decodable order parameters for measurement-induced phases, the details of our specific protocol, and the classical simulation method used to implement it.

A. Review

As discussed in Ref. [14], the entanglement phases may be diagnosed in a scalable way by means of a probe qubit. In the original protocol, the probe qubit is prepared in a maximally-entangled state with the system a time $t = 0$; as time progresses, the monitoring in the system enventually disentangles the probe. However, in the disentangling (area-law) phase, this process takes a short, N-independent amount of time, while in the entangling (volume-law) phase it takes a very long time, diverging exponentially in N. Thus the probe qubit's entanglement at a time $t \sim N$ serves as an order parameter for the transition.

In a given quantum trajectory of the monitored evolution, labelled by the measurement record m, the probe qubit is in a state $\rho_m = (I + \vec{a}_m \cdot \vec{\sigma})/2$. This state is fully described by the Bloch vector, $\vec{a}_m = \mathrm{Tr}(\vec{\sigma}\rho_m)$. The probe qubit's entanglement in trajectory m is given by, e.g., its second Renyi entropy: $S^{(2)} = -\log_2[(1 + a_m^2)/2]$ ($a_m = |\vec{a}_m|$ is the length of the vector). Thus the average length of the Bloch vector, $\mathbb{E}_m[a_m]$, can be used to distinguish the two phases.

Trajectory-averaged observables provide no information about this: indeed, $\mathbb{E}_m \mathrm{Tr}(\rho_m \vec{\sigma}) = \mathbb{E}_m[\vec{a}_m] = \vec{0}$ due to the random direction of the vectors $\vec{a}_m$, independently of their lengths. To access the length of the vectors experimentally, Ref. [14] proposes using a conditional rotation of the probe, U_m, that rotates its Bloch vector along the $+\hat{z}$ direction: $U_m \rho_m U_m^\dagger = (I + a_m \hat{z})/2$. Following this conditional operation, the probe is measured in the computational basis yielding $\mathbb{E}_m[\mathrm{Tr}(U_m \rho_m U_m^\dagger \sigma^z)] = \mathbb{E}_m[a_m]$. Such active feedback would require a classical computation of the conditional operation U_m in real time, which is challenging on present-day quantum simulators due to the short coherence time. To avoid this issue, Ref. [15] uses specially-structured Clifford circuits with pre-compiled feedback operations. We instead develop an approach that works in more general (non-Clifford) circuits, in which the "feedback" step is implemented virtually during a classical post-processing step. In essence, we apply a classical bit flip that on average aligns the z component of $\vec{a}_m$ along $+\hat{z}$; this allows us to extract the average $\mathbb{E}_m[|a_m^z|]$ of the component. Then, using the fact that different instances of the random circuit randomize the direction of $\vec{a}_m$, we connect this to the desired quantity $\mathbb{E}_m[a_m]$, as detailed below.

B. Protocol

Each iteration i of the experiment produces a bitstring $\mathbf{z}^{(i)} = (z_1^{(i)}, \ldots z_N^{(i)})$, with $z_n^{(i)} = \pm 1$ for each qubit in the system (note here we use binary values ± 1 instead of $0, 1$ for convenience). We pick a qubit in the system to act as a "probe", P, and a set of qubits around it to be the "decoding patch", D. Remaining qubits are traced out and not used in the decoding protocol, serving as an "environment" that the probe may get entangled with. We emphasize that the probe qubit P is on the same footing as all other qubits, and can be freely chosen after the experiment, during classical post-processing.

In each iteration of the experiment, we refer to the bits $z_n^{(i)}$ from qubits in the decoding patch D as the *measurement record* $m^{(i)}$, while the bit on the probe qubit is $z_P^{(i)}$. Given the circuit architecture, gates, and a measurement record m, it is possible to classically compute the state $\rho_m^{\rm sim}$ and thus the Bloch vector $\vec{a}_m^{\rm sim}$. Note this step may be computationally hard in general; see Sec. VI C for details on the classical simulation. From each shot of the experiment we define a classical bit, $\tau_m = \mathsf{sign}(\vec{a}_m^{\rm sim} \cdot \hat{z})$ (i.e., $+1$ if $\vec{a}_m^{\rm sim}$ points in the northern hemisphere of the Bloch sphere, -1 otherwise). Finally, we compute the quantity

$$\zeta^{(K)} = \frac{2}{K} \sum_{i=1}^{K} z_P^{(i)} \tau_{m^{(i)}} \tag{S25}$$

where $i = 1, \dots K$ runs over iterations of the experiment. The expected value of ζ after averaging over many realizations of the experiment is

$$\begin{aligned} \zeta^{(K)} \xrightarrow{K\to\infty} \zeta &= 2\sum_m \sum_{z_P=\pm1} \mathsf{Prob}(z_P, m)\, \mathsf{sign}(a_m^{z,\rm sim}) z_P \\ &= 2\sum_m \mathsf{Prob}(m)\, \mathsf{sign}(a_m^{z,\rm sim}) \sum_{z_P=\pm1} \mathsf{Prob}(z_P|m) z_P \\ &= 2\sum_m \mathsf{Prob}(m)\, \mathsf{sign}(a_m^{z,\rm sim}) \sum_{z_P=\pm1} \frac{1+z_P a_m^z}{2} z_P \\ &= 2\sum_m \mathsf{Prob}(m)\, \mathsf{sign}(a_m^{z,\rm sim})\, a_m^z. \end{aligned} \tag{S26}$$

Here $a_m^z = \vec{a}_m \cdot \hat{z}$ denotes the z component of the vector, we have introduced the conditional probability $\mathsf{Prob}(z_P|m) = \mathsf{Prob}(z_p, m)/\mathsf{Prob}(m)$, and used the fact that $\mathsf{Prob}(z_P = \pm 1|m) = \frac{1\pm a_m^z}{2}$. Now, assuming the classical simulation is sufficiently accurate that we can substitute $\mathsf{sign}(a_m^{z,\rm sim}) \mapsto \mathsf{sign}(a_m^z)$, this reduces to

$$\zeta = 2\sum_m \mathsf{Prob}(m) \overbrace{a_m^z \mathsf{sign}(a_m^z)}^{|a_m^z|} = 2\mathbb{E}[|a_m^z|]. \tag{S27}$$

The average of the z component $|a_m^z|$ is correlated with the average length a_m. This relationship can be sharpened by averaging over random circuit realizations, and assuming the direction of $\vec{a}_m$ on the Bloch sphere is uniformly random (this holds if the circuit contains a final layer of random unitary rotations). For a unit vector $\hat{n}$ distributed uniformly on the unit sphere, one has

$$\mathbb{E}[|n^z|] = \frac{1}{2}\int_{-\pi/2}^{\pi/2} \mathrm{d}\theta \sin\theta |\cos\theta| = \frac{1}{2}. \tag{S28}$$

Thus, averaging over circuit realizations U as well as measurement records m, we get $\mathbb{E}[|a_m^z|] = \frac{1}{2}\mathbb{E}[a_m]$, and in conclusion

$$\zeta = \mathbb{E}[a_m]. \tag{S29}$$

We can then define a proxy for the probe's entropy, $S^{(2)}_{\rm proxy} = -\log_2[(1+\zeta^2)/2] = -\log_2[(1+(\mathbb{E}|a_m|)^2)/2]$. Note that this differs from both the "quenched" average of the second Renyi entropy, $S^{(2)} = -\mathbb{E}\log_2[(1+a_m^2)/2]$ and the "annealed" average $S^{(2)} = -\log_2[(1+\mathbb{E}[a_m^2])/2]$. It is true nonetheless that $S^{(2)}_{\rm proxy} = 0$ (1) if and only if the probe qubit is disentangled (maximally entangled) with unit probability.

We decode the same experimental dataset in multiple ways, corresponding to different decoding patches D_r. These are indexed by a *decoding radius* $r = 0, 1, \dots r_{\max}$ such that D_0 is empty, $D_{r_{\max}}$ is the whole system minus the probe, and for each r we have $D_r \subset D_{r+1}$. The patches used for the various system sizes are illustrade in Fig. S14. This defines r-dependent quantities $\zeta(r)$ and $S^{(2)}_{\rm proxy}(r)$. The decoding radius r in our shallow 2D circuits plays a role analogous to time in $1+1$-dimensional monitored circuits: persistence of the probe's entanglement up to large r diagnoses the entangling phase. In addition, it has a natural interpretation in terms of teleportation: by measuring and decoding the outcomes, entanglement may be generated between the probe qubit and the un-measured qubits a distance r away, even if r exceeds the size of the unitary light cone [16]. This point of view further highlights the need to make use of the measurement record, since the measurement-averaged dynamics is strictly causal and cannot give rise to teleportation.

C. Classical simulation algorithm

All the data presented in the main text is obtained from exact simulations of the relevant circuits. While this is expected to become impractical as the circuits approach a beyond-classical regime [1], it is viable for the circuit sizes

studied here ($N \leq 70$ qubits, depth $T = 5$); see Sec. VII for a scalable approach that can overcome this this limitation in one of the phases.

Rather than simulating an N-qubit wavefunction (which becomes prohibitive well below $N = 70$), we simulate "slices" of the circuit by starting from the probe qubit and moving in the direction of increasing r, injecting new qubits and measuring them out as we go, similar to the approach of Ref. [13]. This allows us to obtain the desired quantity $\tau_m = \pm 1$ for an N-qubit experiment without storing in memory a whole N-qubit wavefunction. The classical computational cost is asymptotically $\exp\Big(O(T\sqrt{N})\Big)$, much lower than $\exp(O(N))$ as long as the circuit is shallow (small T). In practice, the precise cost depends sensitively on the choice of probe qubit and decoding patches; in our case, for $N = 70$ with the decoding patches of Fig. S14, it is possible to directly simulate the 1D circuit in Fig. S12 on 24 qubits. However, further optimization of the gate order reduces this to 20 qubits, and presents an algorithm applicable to general geometries and decoding patches, described in detail below.

The algorithm is based on a wavefunction $|\psi\rangle_A$ defined on an "active subsystem" A which always contains the probe P, but otherwise changes over the course of the algorithm: new qubits are introduced in the initial state $|0\rangle$ while other qubits are measured out, i.e. projected on basis states $\langle m_j|$ determined by the experimental measurement record m for the given shot. As it sweeps through the quantum processor, the algorithm sequentially obtains the bits $\tau_m(r)$, with r increasing from 0 to $r_{\max}$; these are then used in conjunction with the experimental measurements z_P to get the r-dependent order parameter $\zeta(r)$. We use S to denote the entire system, i.e. the collection of all qubits in the processor: $S = \{Q_1, \dots Q_N\}$.

1. **Setup**: Choose a probe qubit $P \in S$ and decoding patches $\{D_r\}_{r=0}^{r_{\max}}$.

2. **Initialize**: set the *active subsystem* to $A = \{P\}$, the *initial-state subsystem* to $I = S \setminus P$, the *final-state subsystem* to $F = \emptyset$, the *wavefunction* to $|\psi\rangle_A = |0\rangle_P$

3. **Step** $r = 0$:

   A. Grow:
      (i) obtain the past lightcone* of the probe, $L(P)$
      (ii) add all qubits in $L(P)$ to the active subsystem: $A \leftarrow A \cup L(P)$
      (iii) remove them from the initial-state subsystem: $I \leftarrow I \setminus L(P)$
      (iv) update the wavefunction: $|\psi\rangle_A \leftarrow |\psi\rangle_A \otimes |0\rangle_{L(P)}$

   B. Evolve: apply all allowed gates‡ to $|\psi\rangle_A$.

   C. Collect data: evaluate and store $\tau_m(0) = \mathsf{sign}(\langle\psi|\,\sigma_P^z\,|\psi\rangle)$

   D. Move to next step: $r \leftarrow r + 1$

4. **Steps** $0 < r \leq r_{\max}$:

   A. Grow:
      (i) obtain the past lightcone* $L(Q_n)$ of each qubit $Q_n \in D_r \setminus F$
      (ii) choose qubit Q_{n^*} with $n^* = \mathrm{argmin}_n |L(Q_n) \cap I|$ (i.e. the qubit whose past lightcone* includes the fewest initial-state qubits)
      (iii) add all initial-state qubits in $L(Q_{n^*})$ to the active subsystem: $A \leftarrow A \cup (L(Q_{n^*}) \cap I)$
      (iv) update the wavefunction: $|\psi\rangle_A \leftarrow |\psi\rangle_A \otimes |0\rangle_{L(Q_{n^*})\cap I}$
      (v) remove the qubits from the initial-state subsystem: $I \leftarrow I \setminus L(Q_{n^*})$

   B. Evolve: apply all allowed gates‡ to $|\psi\rangle_A$.

   C. Project: for any qubit $Q_j \in A \cap D_r$ with no gates left to apply,
      (i) remove it from the active subsystem, $A \leftarrow A \setminus \{Q_j\}$
      (ii) add it to the final-state subsystem, $F \leftarrow F \cup \{Q_j\}$
      (iii) update the wavefunction by projecting according to the experimental measurement outcome m_j: $|\psi\rangle_A \leftarrow \langle m_j|\psi\rangle_A / \|\langle m_j|\psi\rangle_A\|$

   D. If $D_r \subseteq F$ (all qubits in the decoding patch D_r have been measured out),
      (i) Collect data: evaluate and store $\tau_m(r) = \mathsf{sign}(\langle\psi|\,\sigma_P^z\,|\psi\rangle)$
      (ii) Move to next step: $r \leftarrow r + 1$

   D'. Else: go to step 4A.

5. **End** when $r > r_{\max}$. Return values of $\{\tau_m(r)\}_{r=0}^{r_{\max}}$.

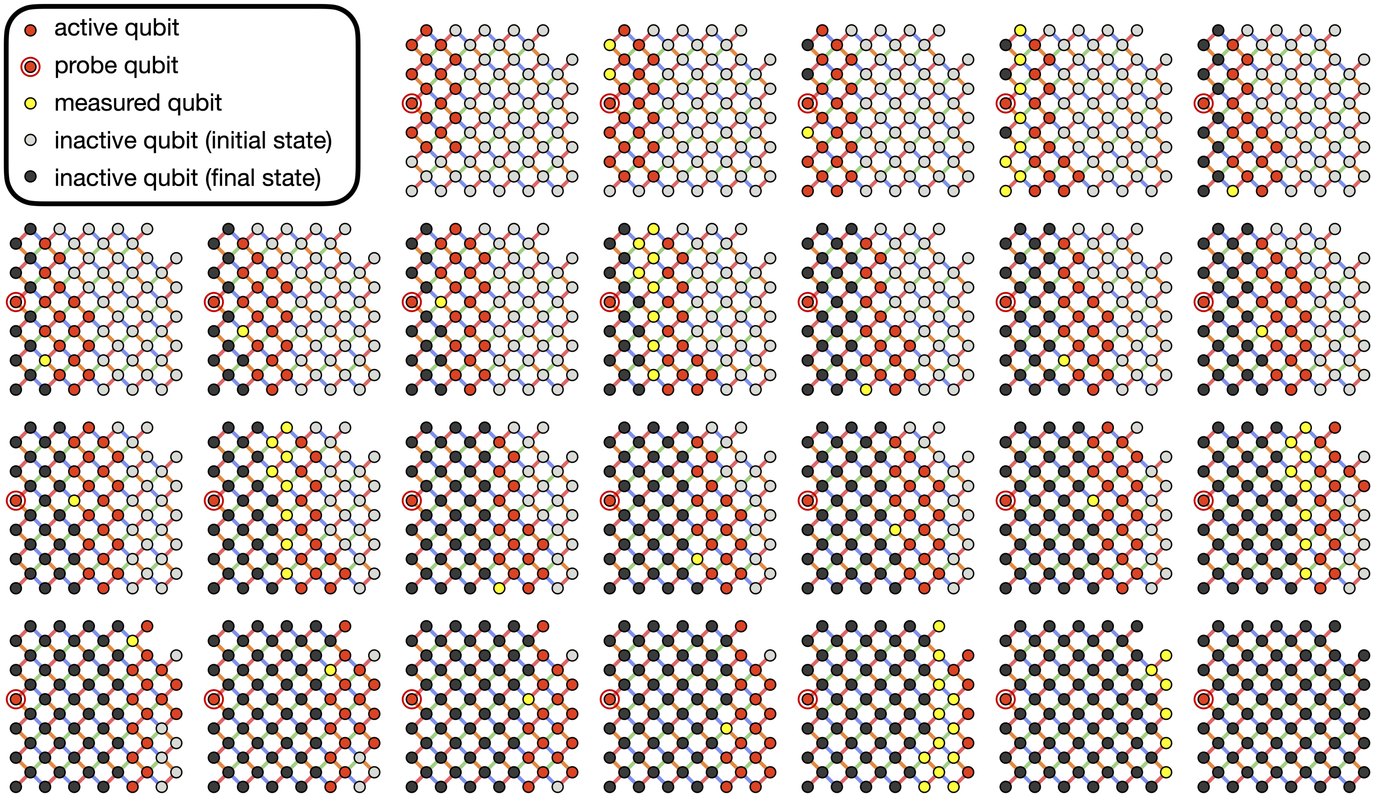

FIG. S15. Classical simulation algorithm for quantum circuits on $N = 70$ qubits (depth $T = 5$). The active subsystem A is indicated in red. It always includes the probe P (circled qubit). Light gray qubits make up the initial-state subsystem I (not yet part of the wavefunction), black qubits are the final-state subsystem F (already projected out and not part of the wavefunction anymore). Yellow qubits are projected out right before the evolution step in each panel. The active subsystem sweeps the chip from left to right and the simulation terminates when all qubits (except the probe) have been measured out. At each step in the simulation, the active subsystem contains at most 20 qubits.

Definitions used in the algorithm:

* Past lightcone: the past lightcone $L(Q_n)$ of a qubit Q_n is the subset of qubits that can be reached starting from Q_n at the final time $t = T$ and hopping along gates in the circuit for times $t = T, T-1, \ldots 1$. It is the minimal subsystem needed to simulate the state (reduced density matrix) of qubit Q_n at the end of the circuit.

‡ Allowed gates: a gate acting on qubits Q_i, Q_j in the first layer of the circuit ($t = 1$) is *allowed* if both qubits $Q_{i,j}$ belong to the active subsystem A. Gates in following layers $t = 2, \ldots T$ are allowed if the same criterion is met *and* all gates on $Q_{i,j}$ in layers $\tau < t$ have already been applied.

The algorithm splits the system S into subsystems I (initial), A (active) and F (final). All qubits (except the probe) start in I, move to A and end in F. When F coincides with a decoding patch D_r, we can evaluate the classical bit $\tau_m(r)$ needed for decoding. A concrete instance of the algorithm is shown in Fig. S15 for the largest circuit decoded in this work ($N = 70$ qubits, depth $T = 5$, decoding patches $\{D_r : 0 \leq r \leq r_{\max} = 12\}$ as in Fig. S14). The color coding shows which qubits are measured out before each step, in addition to the three subsystems. Note that in several cases, after a patch D_r is complete, we may immediately measure out some of the qubits in D_{r+1}; in these cases, Fig. S15 combines the two steps in a single panel.

Finally, we note that step 4.A(ii) in the algorithm corresponds to a greedy optimization of the tensor contraction order. While this works well in practice for the circuits studied here, in principle one may use better optimization strategies to push this decoding approach close to the beyond-classical frontier.

D. Simulated decoding data

In the process of decoding experimental data as explained above, one obtains classically-simulated values of the probe qubit's Bloch vector z-component, $a_m^{z,\mathrm{sim}}$. These may be used to compute a simulated order parameter ζ^{sim} by

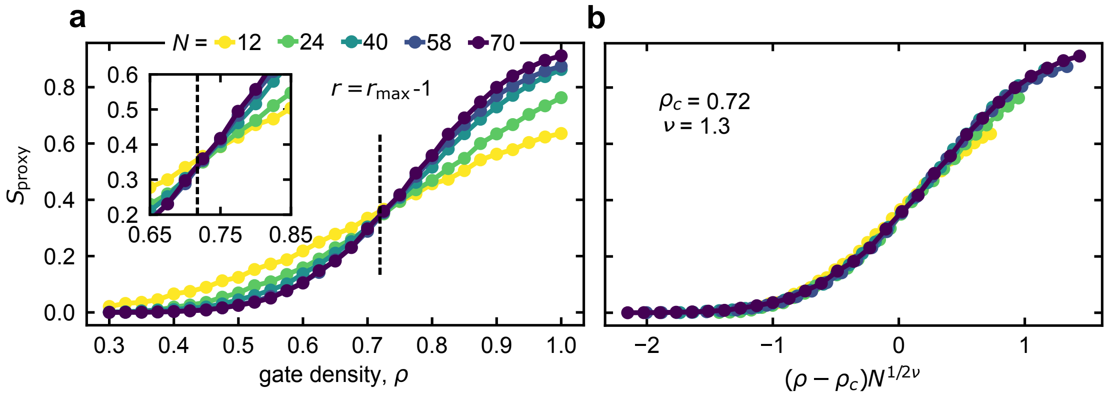

FIG. S16. Simulated decoding of probe qubit (cf. Fig. 4 in the main text). (a) Simulated proxy entropy $S_{\rm proxy}$ at decoding radius $r = r_{\rm max} - 1$ as a function of ρ for all system sizes studied. Vertical dashed line indicates the estimated $\rho_c = 0.72$. Inset: zoomed-in finite-size crossing. (b) Scaling collapse of the data vs $(\rho - \rho_c)N^{1/(2\nu)}$ with $\rho_c = 0.72$ and $\nu = 1.3$.

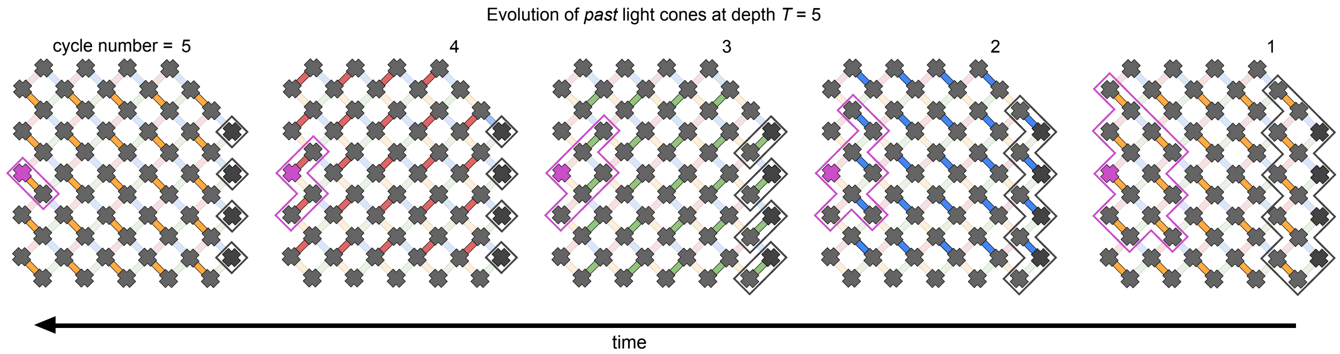

FIG. S17. Determination of the past light cones of the probe qubit (pink) and qubits along the right-most column (black) of the $N = 58$ geometry. We begin with the final cycle of two qubit operations at depth $T = 5$ and calculate the past light cone by going backward in time until we reach the first cycle of the circuit. The two subsystems have non-overlapping past light cones and are therefore causally disconnected.

replacing a^z_m (the "true", experimental value) with $a^{z,{\rm sim}}_m$ (the simulated value) in Eq. (S26):

$$\zeta^{(K),{\rm sim}} = \frac{2}{K}\sum_{i=1}^{K}|a^{z,{\rm sim}}_{m^{(i)}}| \quad \xrightarrow{K\to\infty} \quad \zeta^{\rm sim} = 2\mathbb{E}[|a^{z,{\rm sim}}_m|] \tag{S30}$$

This simulated order parameter provides information about the ideal (noiseless) dynamics. In particular it provides evidence of a phase transition in the ideal model, with finite-size crossing behavior, and also provides an estimate of the location of the phase transition. Data for $S^{\rm sim}_{\rm proxy}$, obtained from the same circuits as Fig. 4 of the main text, is shown in Fig. S16(a). A finite-size crossing at $\rho = \rho_c \simeq 0.72$ is visible. The numerically well-established value of the correlation length critical exponent [17], $\nu \simeq 1.3$, yields a reasonable scaling collapse of the different sizes, Fig. S16(b). This is indicative of a measurement-induced phase transition into the teleporting phase [16] above ρ_c. In the teleporting phase quantum information (aided by classical communication) travels faster than the limits imposed by locality and causality of unitary dynamics. Indeed in the unitary circuit the probe and unmeasured qubits are causally disconnected, with non-overlapping past light cones. This means that there is no qubit in the initial state that may share information with both of them. The formation of the past light cones of the probe (pink) and unmeasured qubits (black) as a function of the cycle number at depth $T = 5$ is illustrated in Fig. S17.

VII. Approximate decoding

Here we describe an approximate decoding algorithm whose computational time scales polynomially in system size N, as opposed to the decoding protocol based on exact simulation in Sec. VI. This efficient scaling means the algorithm could be applied on larger quantum processors in the beyond-classical regime. The algorithm succeeds in the disentangling phase, but fails in the entangling phase. While the protocol's failure is in and of itself a diagnostic of the phase transition, the development of scalable approaches that succeed in both phases and near the critical point in generic models remains an important open problem [14, 18–20].

A. MPS method

We adopt the same general approach of Sec. VI, except the computation of τ_m is implemented approximately via a matrix product state (MPS) method [21]. MPSs are one-dimensional structures characterized by a cut-off χ, the *bond dimension*, that caps the amount of entanglement across each bond: $S \leq \log_2(\chi)$. Classical computational cost scales polynomially in χ, thus exponentially in S (for an exact simulation). In the disentangling phase, the entanglement within the quasi-1D subsystems used in the simulation is predicted to obey an area-law, $S = O(1)$. Therefore an MPS representation with sufficiently large but finite χ is expected to provide accurate results even in the $N \to \infty$ limit. Conversely, in the entangling phase, we expect S to grow with decoding radius r, $S \sim r$, up to a maximum value $S \sim \sqrt{N}$ (proportional to the linear size of the quasi-1D subsystem). Thus the bond dimension needed for accurate simulation grows super-polynomially with N and accurate simulation becomes unfeasible.

We simulate the 1D monitored circuit of Fig. S12(b) with an MPS truncated to maximum bond dimension χ (variable). The circuit comprises local unitary gates, projective measurements, and resetting to the $|0\rangle$ state, all operations that can be performed straightforwardly on MPSs. The probe qubit is kept at an end of the MPS throughout the simulation. Note that the spatial range of gates is $\propto T$, thus the method relies on the circuit being shallow (i.e. T being finite).

B. Results

Results of the approximate decoding algorithm are shown in Fig. S18 at values of ρ across the phase diagram and for various bond dimensions χ. In the disentangling phase, with moderate bond dimension $\chi \leq 256$ the decoding algorithm is fully-converged for $\rho = 0.3$ and nearly-converged for $\rho = 0.5$ (e.g. reasonable extrapolation to $\chi = \infty$ is possible). Near the critical point ($\rho = 0.7$) the accuracy of the simulation visibly deteriorates with increasing r, as significant truncation errors build up in the simulation. In particular, while $\zeta(r)$ is expected to monotonically increase with r, it is found to decrease at large r. As a result the mitigated entropy $\tilde{S}_{\text{proxy}}(r)$ acquires unphysical negative values. Finally, deep in the entangling phase ($\rho = 1$) the approximate decoding algorithm fails completely, $\zeta(r) \ll 1$ for all r, with no visible improvement at these values of χ.

C. Effect of classical simulation errors

In general, the simulated Bloch vector $\vec{a}_m^{\text{sim}}$ will not be perfectly identical to the true (experimental) one $\vec{a}_m$. This is due to unavoidable noise and errors in the experiment, but in the case of approximate decoding may also arise from uncontrolled truncation errors in the classical simulation. Let us crudely model these effects by assuming that $\tau_m = \mathsf{sign}(a_m^{z,\text{sim}})$ is correct (i.e. equal to $+\mathsf{sign}(a_m^z)$) with probability q and incorrect (i.e. equal to $-\mathsf{sign}(a_m^z)$) otherwise, independently of m and circuit realization. Then Eq. (S27) yields

$$\zeta_{\text{approx}} = 2\mathbb{E}\left[a_m^z \mathsf{sign}(a_m^{z,\text{sim}})\right] = 2(2q-1)\mathbb{E}[|a_m^z|] = (2q-1)\zeta. \tag{S31}$$

The net result is that the decoded signal is damped by a factor $(2q-1) \leq 1$. Further including r-dependence in q (as both quantum measurement and classical simulation errors build up with increasing r) gives $\zeta_{\text{approx}}(r) \sim (2q(r)-1)\zeta(r)$, distorting the signal and causing the error mitigation to fail. This explains the behavior of Fig. S18 in the entangling phase ($\rho = 1$), where decoding fails.

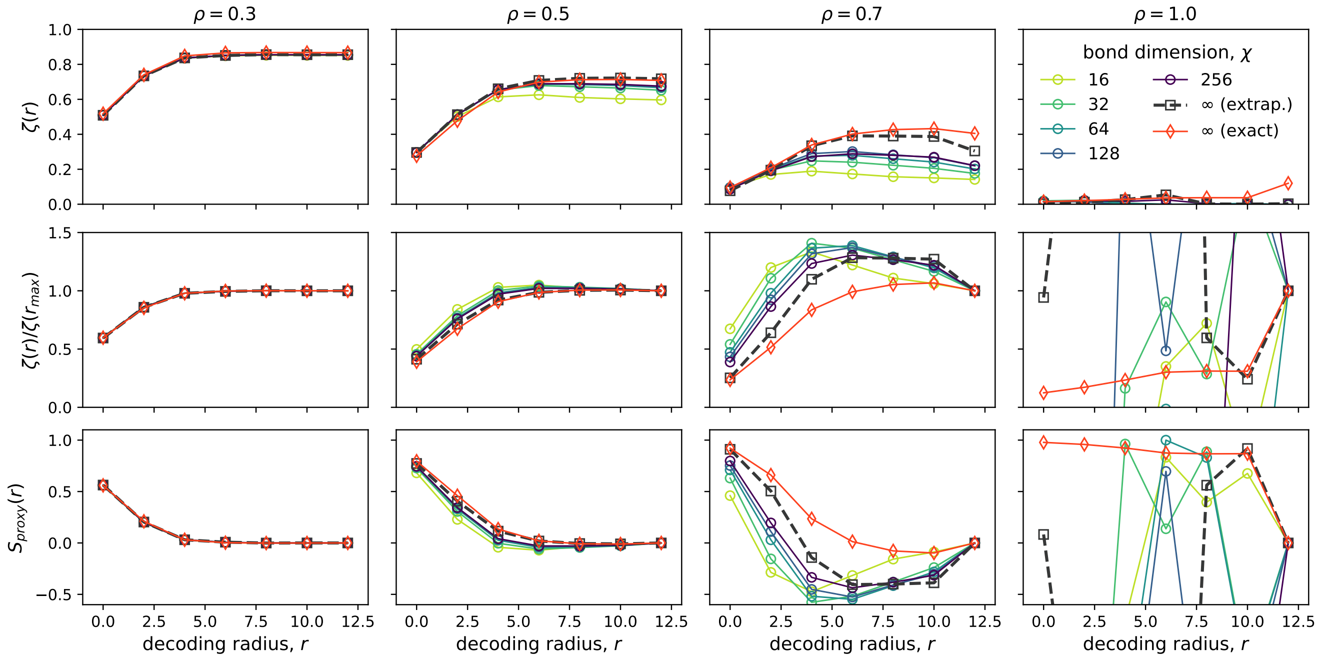

FIG. S18. Results of approximate decoding of $N = 70$ experimental data based on MPS with bond dimension $\chi = 16, \dots 256$, at four values of ρ across the phase diagram. Top row: decoded order parameter ζ as a function of decoding radius r. Middle row: error-mitigated order parameter $\tilde{\zeta}(r) = \zeta(r)/\zeta(r_{\max})$. Bottom row: error-mitigated proxy entropy $\tilde{S}_{\text{proxy}}(r) = -\log_2[(1 + \tilde{\zeta}(r)^2)/2]$. Along with the fixed-$\chi$ data (circles), we show an extrapolation to $\chi \to \infty$ (squares) and a comparison with the results of decoding based on exact simulation (diamonds) as in Sec. VI. The extrapolation is obtained from fits to an empirical ansatz $\alpha/\log(\chi) + \beta$ on $\chi = 32, \dots 256$.

† Google Quantum AI and Collaborators

J. C. Hoke[1,2,*], M. Ippoliti[2,3,*], E. Rosenberg[1,4], D. Abanin[1], R. Acharya[1], T. I. Andersen[1], M. Ansmann[1], F. Arute[1], K. Arya[1], A. Asfaw[1], J. Atalaya[1], J. C. Bardin[1,5], A. Bengtsson[1], G. Bortoli[1], A. Bourassa[1], J. Bovaird[1], L. Brill[1], M. Broughton[1], B. B. Buckley[1], D. A. Buell[1], T. Burger[1], B. Burkett[1], N. Bushnell[1], Z. Chen[1], B. Chiaro[1], D. Chik[1], J. Cogan[1], R. Collins[1], P. Conner[1], W. Courtney[1], A. L. Crook[1], B. Curtin[1], A. G. Dau[1], D. M. Debroy[1], A. Del Toro Barba[1], S. Demura[1], A. Di Paolo[1], I. K. Drozdov[1,6], A. Dunsworth[1], D. Eppens[1], C. Erickson[1], E. Farhi[1], R. Fatemi[1], V. S. Ferreira[1], L. F. Burgos[1] E. Forati[1], A. G. Fowler[1], B. Foxen[1], W. Giang[1], C. Gidney[1], D. Gilboa[1], M. Giustina[1], R. Gosula[1], J. A. Gross[1], S. Habegger[1], M. C. Hamilton[1,7], M. Hansen[1], M. P. Harrigan[1], S. D. Harrington[1], P. Heu[1], M. R. Hoffmann[1], S. Hong[1], T. Huang[1], A. Huff[1], W. J. Huggins[1], S. V. Isakov[1], J. Iveland[1], E. Jeffrey[1], Z. Jiang[1], C. Jones[1], P. Juhas[1], D. Kafri[1], K. Kechedzhi[1], T. Khattar[1], M. Khezri[1], M. KieferovÃ¡[1,8], S. Kim[1], A. Kitaev[1], P. V. Klimov[1], A. R. Klots[1], A. N. Korotkov[1,9], F. Kostritsa[1], J. M. Kreikebaum[1], D. Landhuis[1], P. Laptev[1], K.-M. Lau[1], L. Laws[1], J. Lee[1,10], K. W. Lee[1], Y. D. Lensky[1], B. J. Lester[1], A. T. Lill[1], W. Liu[1], A. Locharla[1], O. Martin[1], J. R. McClean[1], M. McEwen[1], K. C. Miao[1], A. Mieszala[1], S. Montazeri[1], A. Morvan[1], R. Movassagh[1], W. Mruczkiewicz[1], M. Neeley[1], C. Neill[1], A. Nersisyan[1], M. Newman[1], J. H. Ng[1], A. Nguyen[1], M. Nguyen[1], M. Y. Niu[1], T. E. O'Brien[1], S. Omonije[1], A. Opremcak[1], A. Petukhov[1], R. Potter[1], L. P. Pryadko[1,11], C. Quintana[1], C. Rocque[1], N. C. Rubin[1], N. Saei[1], D. Sank[1], K. Sankaragomathi[1], K. J. Satzinger[1], H. F. Schurkus[1], C. Schuster[1], M. J. Shearn[1], A. Shorter[1], N. Shutty[1], V. Shvarts[1], J. Skruzny[1], W. C. Smith[1], R. Somma[1], G. Sterling[1], D. Strain[1], M. Szalay[1], A. Torres[1], G. Vidal[1], B. Villalonga[1], C. V. Heidweiller[1], T. White[1], B. W. K. Woo[1], C. Xing[1], Z. J. Yao[1], P. Yeh[1], J. Yoo[1], G. Young[1], A. Zalcman[1], Y. Zhang[1], N. Zhu[1], N. Zobrist[1], H. Neven[1], R. Babbush[1], D. Bacon[1], S. Boixo[1], J. Hilton[1], E. Lucero[1], A. Megrant[1], J. Kelly[1], Y. Chen[1], V. Smelyanskiy[1], X. Mi[1], V. Khemani[2], P. Roushan[1]

1 Google Research, Mountain View, CA, USA

2 Department of Physics, Stanford University, Stanford, CA, USA

3 Department of Physics, University of Texas at Austin, Austin, TX, USA

4 Department of Physics, Cornell University, Ithaca, NY, USA

5 Department of Electrical and Computer Engineering, University of Massachusetts, Amherst, MA, USA

6 Department of Physics, University of Connecticut, Storrs, CT

7 Department of Electrical and Computer Engineering, Auburn University, Auburn, AL, USA

8 QSI, Faculty of Engineering & Information Technology, University of Technology Sydney, NSW, Australia

9 Department of Electrical and Computer Engineering, University of California, Riverside, CA, USA

10 Department of Chemistry, Columbia University, New York, NY, USA

11 Department of Physics and Astronomy, University of California, Riverside, CA, USA

∗ These authors contributed equally to this work.

---

[1] F. Arute *et al.*, Quantum supremacy using a programmable superconducting processor, Nature **574**, 505 (2019).

[2] A. Morvan *et al.*, Formation of robust bound states of interacting microwave photons, Nature **612**, 240 (2022).

[3] B. Foxen *et al.* (Google AI Quantum), Demonstrating a continuous set of two-qubit gates for near-term quantum algorithms, Phys. Rev. Lett. **125**, 120504 (2020).

[4] M. P. Harrigan *et al.*, Quantum approximate optimization of non-planar graph problems on a planar superconducting processor, Nature Physics **17**, 332 (2021).

[5] T. Anderson *et al.*, Non-abelian braiding of graph vertices in a superconducting processor, Nature **618**, 264 (2023).

[6] D. Jafferis *et al.*, Traversable wormhole dynamics on a quantum processor, Nature **612**, 51 (2022).

[7] S. J. van Enk and C. W. J. Beenakker, Physical Review Letters **108**, 10.1103/physrevlett.108.110503 (2012).

[8] A. Elben, B. Vermersch, M. Dalmonte, J. Cirac, and P. Zoller, Rényi entropies from random quenches in atomic hubbard and spin models, Physical Review Letters **120**, 10.1103/physrevlett.120.050406 (2018).

[9] T. Brydges *et al.*, Probing Rényi entanglement entropy via randomized measurements, Science **364**, 260 (2019).

[10] K. Satzinger *et al.*, Realizing topologically ordered states on a quantum processor, Science **374**, 1237 (2021).

[11] Y. Li, S. Vijay, and M. P. Fisher, Entanglement domain walls in monitored quantum circuits and the directed polymer in a random environment, PRX Quantum **4**, 10.1103/prxquantum.4.010331 (2023).

[12] M. Ippoliti and V. Khemani, Postselection-free entanglement dynamics via spacetime duality, Physical Review Letters **126**, 060501 (2021).

[13] J. C. Napp *et al.*, Efficient classical simulation of random shallow 2d quantum circuits, Phys. Rev. X **12**, 021021 (2022).

[14] M. J. Gullans and D. A. Huse, Scalable probes of measurement-induced criticality, Phys. Rev. Lett. **125**, 070606 (2020).

[15] C. Noel *et al.*, Observation of measurement-induced quantum phases in a trapped-ion quantum computer, Nature Physics **18**, 760 (2022).

[16] Y. Bao *et al.*, Finite time teleportation phase transition in random quantum circuits, arXiv preprint arXiv:2110.06963 (2021).

[17] A. Zabalo, M. J. Gullans, J. H. Wilson, S. Gopalakrishnan, D. A. Huse, and J. H. Pixley, Critical properties of the measurement-induced transition in random quantum circuits, Phys. Rev. B **101**, 060301 (2020).

[18] H. Dehghani, A. Lavasani, M. Hafezi, and M. J. Gullans, Neural-network decoders for measurement induced phase transitions, Nature Communications **14**, 10.1038/s41467-023-37902-1 (2023).

[19] Y. Li, Y. Zou, P. Glorioso, E. Altman, and M. P. Fisher, Cross entropy benchmark for measurement-induced phase transitions, Physical Review Letters **130**, 10.1103/physrevlett.130.220404 (2023).

[20] F. Barratt *et al.*, Transitions in the learnability of global charges from local measurements, Phys. Rev. Lett. **129**, 200602 (2022).

[21] U. Schollwock, The density-matrix renormalization group in the age of matrix product states, Annals of Physics **326**, 96 (2011), january 2011 Special Issue.
